# Securing Cloud-Assisted Connected and Autonomous Vehicles: An In-Depth Threat Analysis and Risk Assessment

**DOI:** 10.3390/s24010241

**Published:** 2023-12-31

**Authors:** Al Tariq Sheik, Carsten Maple, Gregory Epiphaniou, Mehrdad Dianati

**Affiliations:** Warwick Manufacturing Group (WMG), University of Warwick, Coventry CV4 7AL, UK; cm@warwick.ac.uk (C.M.); gregory.epiphaniou@warwick.ac.uk (G.E.); m.dianati@warwick.ac.uk (M.D.)

**Keywords:** threat analysis, threat modeling, risk assessment, cyber security, connected vehicles, autonomous vehicles, edge computing, cloud, taxonomy, attack tree, countermeasures

## Abstract

As threat vectors and adversarial capabilities evolve, Cloud-Assisted Connected and Autonomous Vehicles (CCAVs) are becoming more vulnerable to cyberattacks. Several established threat analysis and risk assessment (TARA) methodologies are publicly available to address the evolving threat landscape. However, these methodologies inadequately capture the threat data of CCAVs, resulting in poorly defined threat boundaries or the reduced efficacy of the TARA. This is due to multiple factors, including complex hardware–software interactions, rapid technological advancements, outdated security frameworks, heterogeneous standards and protocols, and human errors in CCAV systems. To address these factors, this study begins by systematically evaluating TARA methods and applying the Spoofing, Tampering, Repudiation, Information disclosure, Denial of service, and Elevation of privileges (STRIDE) threat model and Damage, Reproducibility, Exploitability, Affected Users, and Discoverability (DREAD) risk assessment to target system architectures. This study identifies vulnerabilities, quantifies risks, and methodically examines defined data processing components. In addition, this study offers an attack tree to delineate attack vectors and provides a novel defense taxonomy against identified risks. This article demonstrates the efficacy of the TARA in systematically capturing compromised security requirements, threats, limits, and associated risks with greater precision. By doing so, we further discuss the challenges in protecting hardware–software assets against multi-staged attacks due to emerging vulnerabilities. As a result, this research informs advanced threat analyses and risk management strategies for enhanced security engineering of cyberphysical CCAV systems.

## 1. Introduction

Cloud-Assisted Connected and Autonomous Vehicles (CCAVs) are at the forefront of vehicular technology, integrating cloud services, edge computing, Roadside Units (RSU), and various Connected and Autonomous Vehicle (CAV) models [1,2,3]. Operating on complex hardware and software platforms, these systems are the subject of ongoing research aimed at bolstering security and safety. With rapid technological evolution, CCAVs face heightened security risks, particularly from threats that can compromise security requirements like confidentiality, integrity, availability, authenticity, authorization, and accountability [4,5,6]. This paper focuses on delineating these security threats, encompassing both targeted and multistaged attacks on the hardware and software systems of CCAVs. This study systematically identifies and analyses complex threats, conducts an in-depth risk assessment, and formulates comprehensive countermeasures [7]. The aim is to enhance understanding of these emerging threats and contribute to the development of robust security strategies for CCAV systems.

A prior system-centric survey of the CCAV threat landscape using a platooning use case has led to formulating and mapping an attack taxonomy [8]. The survey identified 132 threats from the literature, 64 real-life security breaches, and 22 threats specific to platooning microservices. The results highlight limitations, open challenges, and the need for future work that implements threat analysis and risk assessment (TARA) methods in the broader CCAV ecosystem. TARA methods provide a systematic approach to modeling CCAVs [9], which aid the identification of strengths and weaknesses in their systems by assessing their impact.

Research on reference architectures for CCAVs, particularly regarding their implementation and operation with edge/cloud and cloud environments, is in the early stages [4,5]. Although an increasing number of theoretical, lab-based, and real-world attacks are known, these have not been considered or analyzed in the CCAV context but have been surveyed in [8]. As a result, it is important to contribute to this field by formulating a research-based reference architecture for CCAVs to systematically perform TARA in order to capture the threats exposed to the assets in the system.

This study aims to systematically examine and quantify risks to CCAV systems and explore the issues associated with securing CCAVs effectively. This was achieved through completing the following objectives: (1) analyze architectures for CCAVs; (2) perform a systematic threat analysis and risk assessment to evaluate the impact on trust domains and security requirements; and (3) suggest countermeasures on trust domains using a defense taxonomy by mapping hardware and software components of CCAV systems.

The remainder of this paper is organized in the following manner: Section 2 presents an overview of the three-tier architecture system, offering contextual information. In Section 3, an examination of the TARA methods is conducted to facilitate the comparison and analysis of different approaches. In Section 4, an adversarial model is presented, which examines the motivations, capabilities, and opportunities of various threat actors in the context of developing threats and evaluating their associated risks. In this study, Section 5 provides an in-depth description of the research methodology used. Section 6 of the document focuses on the examination of the TARA. This section provides a comprehensive overview of the system architecture, the outcomes of the STRIDE/DREAD analysis, the trust domains that are affected, and the security requirements that arise from these findings. Section 7 covers an examination and discourse on the identified threats, vulnerabilities, and implications on security requirements, accompanied by a discussion of the constraints inherent in the methodology, resulting in the formulation of an attack tree. Section 8 of the document places emphasis on countermeasures, organizing them into distinct categories using a defense taxonomy and subsequently offering a comprehensive analysis. In conclusion, Section 9 serves as the concluding section of this research paper.

## 2. Background

**Figure 1:**
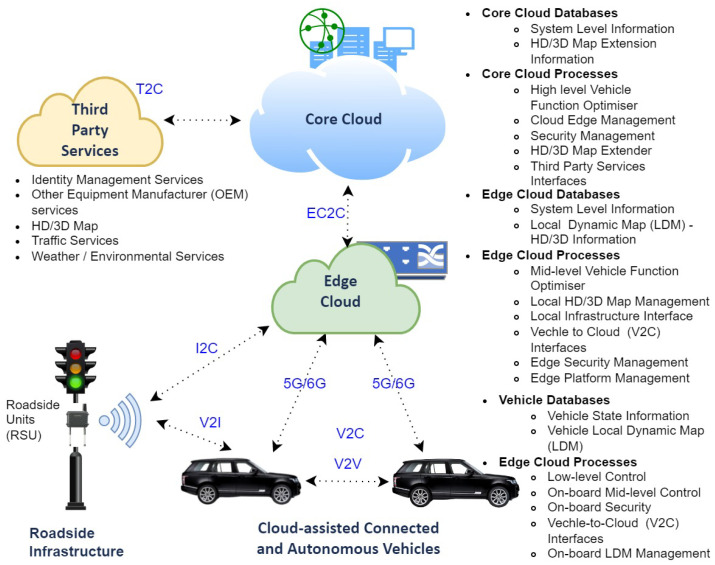
High-level view of Cloud-Assisted Connected and Autonomous Vehicles, adapted from [5,10,11].

CAVs require real-time data exchange and processing, and delays incurred by limited onboard computing hardware capabilities are potentially dangerous. A notable advancement in this area has been Cloud-Assisted Real-Time Methods for Autonomy (CARMA), a project financed by the EPSRC and Jaguar Land Rover. CARMA uses an Internet-of-Things (IoT)-inspired three-tier architecture (see Figure 1), suitable for mission-critical and time-sensitive applications. Each tier has different functions: (i) Tier 1—CCAVs: These vehicles are designed to process data locally while maintaining communication with edge clouds or RSUs through Cooperative Awareness Messages (CAMs); (ii) Tier 2—Core Cloud; The core cloud handles the computing for mission planning, mobile infrastructure management, security and database management, map management, and third-party applications, while also offering services to the edge cloud; and (iii) Tier 3—Edge Cloud: The edge cloud performs off-board vehicular computation, regional map analysis, and security algorithms such as authentication. By leveraging powerful infrastructures like the core cloud and third-party services, the edge cloud can execute latency-free localized computations for CCAVs; however, this increases the attack surface.

## 3. Threat Analysis and Risk Assessment Methods

TARA is a systematic technique used to model CCAV applications and trust domains and mitigate risks in these systems through a thorough and valid evaluation of the current state of the system [12]. The complexity of CCAV ecosystems makes them vulnerable to targeted multistage cyberattacks, which can have a significant impact on both hardware and software components. The TARA considers this challenge of identifying and understanding attack paths to effectively deploy appropriate safeguards. The TARA may employ formal techniques such as data flow diagrams (DFDs), attack trees, MITRE ATT&CK, tactics, techniques, and procedures to analyze threats. Alternatively, they may adopt methods explored in literature, including advanced techniques such as discrete-time Markov chains, state-space models, and Bayesian networks; however, the latter is not adopted in this research due to the evolving nature of the landscape. As such, this research used the data collected from literature and real-life incidents from previous research available in [8]. On the basis of these data, this research considered the following approaches to perform the TARA.

### 3.1. Microsoft’s STRIDE/DREAD

The widely adopted STRIDE technique, developed by Microsoft as part of the Security Development Lifecycle, serves to identify, describe, and analyze threats, their impacts, as well as entry points and vectors on the trust domains of the system [9]. It benefits organizations by allowing them to respond to changes throughout the lifespan of the system. The term STRIDE corresponds to (1) Spoofing (S)—the process of forging the identity of a person or a system. It may be directed towards a configuration, a file, a machine, sensory data or system, or a person’s specific function. Spoofing compromises authenticity. (2) Tampering (T)—the process of modifying data to induce an error in the system’s functioning. It may be directed at individual files, sensory data, or whole networks and compromises integrity. (3) Repudiation (R)—a method of eradicating traces of system activity related to log files. Repudiation compromises non-repudiation. (4) Information Disclosure (I)—the process of gaining unauthorized access to the data storage or data flow. This compromises confidentiality. (5) Denial of Service (D)—the process of interfering with or disturbing normal functioning. Denial of Service compromises availability. (6) Elevation of Privilege (E)—the process of performing an unauthorized action in the system, compromising access and authorization.

STRIDE can be adapted to CyberPhysical Systems (CPSs) by deconstructing them into logical and physical components, considering the interplay of internal and external units. This helps to develop DFDs for each of these components. It adds authenticity, nonrepudiation, safety, and authorization to the standard CIA (Confidentiality, Integrity, and Availability) [13].

DREAD was created to assist STRIDE in risk assessment [6]. It presents a categorisation-based technique for assessing risks using Equation (1) and the adversarial model discussed in Section 4, which is based on (1) Damage Potential (D)—the assessment of the damage inflicted on a system by a cyberattack. (2) Reproducibility (R)—the assessment of the means through which a cyberattack may be replicated. For example, if an assault can be repeated, it poses a serious danger to the system. (3) Exploitability (E)—the assessment of the feasibility of conducting a cyberattack in comparison to the requirements for successful execution. (4) Affected Users (A)—the evaluation that takes into account the potential effect of the attack on the number of users. (5) Discoverability (D)—the examination that takes into account the attack’s discoverability inside the system.

(1) Risk=(D+R+E+A+D)/5

### 3.2. Operationally Critical Threat, Asset, and Vulnerability Evaluation (OCTAVE)

OCTAVE is a risk-based technique developed by the Software Engineering Institute, the CERT Division [14,15]. OCTAVE has two broad methods. These are OCTAVE-S and OCTAVE Allegro. OCTAVE focuses on mitigating organizational risks with interdisciplinary approaches, including senior executives, operational managers, and security professionals. The process is divided into three stages: (i) establishing asset-based threat profiles for organizational security assessment; (ii) identification of infrastructure vulnerabilities; and (iii) designing a cybersecurity plan based on the identified threats to important assets.

### 3.3. Process for Attack Simulation and Threat Analysis (PASTA)

The PASTA considers security requirements to identify the most credible threats to a system while balancing and adhering to business goals. It provides a systematic framework that includes creating detailed documentation of the considered system. This can be labor-intensive compared with other threat modeling methods and may be challenging for developers to understand. The process involves (i) defining business and security goals and the impact of security measures on the organization, (ii) defining the technological scope, (iii) decomposition of system security, (iv) creating DFDs, (v) evaluating threats based on the security decomposition and diagrams, (vi) assessing system vulnerabilities and weaknesses, (vii) modeling potential cyberattacks, and (viii) evaluating the resulting risks and their impact on business [16].

### 3.4. Composite Threat Modeling

The US Department of Transportation and National Highway Traffic Safety Administration created the Composite Threat Modeling approach exclusively for vehicles that are connected and/or autonomous [17]. This methodology is divided into two stages: (i) identifying important components and (ii) analyzing the respective threats to those components. This enables security measures to be tailored based on the criticality of the threat. The technique demands that DFDs be represented with all physical or networked components, entry/exit points, and data formats. Following this, threats may be recognized by analyzing the DFDs with the purpose of identifying (i) critical data flows needed for the mission; (ii) direct/indirect data flow that may affect a critical component; (iii) the components changing the data in the network; (iv) the physical/wireless threat entry points; and (v) the security properties of the system.

### 3.5. Attack Tree

The ability to detect, assess, and visualize threats is crucial for CCAVs. In terms of complexity, it can be difficult to fully understand the intricacies involved in an attack pathway. To tackle this issue, researchers have suggested using attack trees to visually represent cyber attacks. These representations offer a comprehensive view of the steps and components involved in a cyber attack. Despite their benefits, there is currently a lack of consistency in how attack trees are depicted. To enhance their effectiveness, it is essential to establish a unified representation. Therefore, while attack trees can be very useful in visualizing threats in CCAVs, they must be standardized for the improved perception, understanding, and representation of detected threats from CCAVs [18].

### 3.6. Analysis of TARA Techniques

We considered five potentially relevant threat modeling methods for a CCAV ecosystem [19]. It is difficult to address all the challenges of such a use case with a single solution [20]. Therefore, a selection of metrics developed from the work in [21] were used to assess the methods in this research: (i) Maturity: Is the technique well-defined and has it been employed in earlier research? (ii) Adaptability: Is the technique adaptable to the unique needs of the use case? (iii) Safety and Security Dependency Coverage: Is the technique inclusive of the implications of safety and security? (iv) Hardware and Software Threats: Does the analysis include both hardware and software threats? (v) Documentation: Does the technique have an extensive documentation?

Evaluating various methodologies for cyber threat detection enables objective analysis, highlighting their respective strengths and weaknesses. Table 1 summarizes the considerations based on the defined metrics. Comparisons are drawn between Attack Tree, Composite Threat Modeling, PASTA, OCTAVE, and STRIDE/DREAD. Composite Threat Modeling, PASTA, and STRIDE/DREAD utilize DFDs in their frameworks, which is helpful in analyzing attack paths and affected components in CCAVs. STRIDE/DREAD, PASTA, and Attack Trees demonstrate higher adaptability for new use cases and both are capable of capturing threats from reference architecture. However, PASTA requires extensive organizational consultation. Thus, STRIDE/DREAD and Attack Tree are followed for the rest of this study. The following section discusses the considered adversarial model, research methodology, results, and analysis.

## 4. Adversarial Model

Adversaries exploit vulnerabilities for a variety of reasons and incentives; see Table 2. An attacker may be aggressive or passive, external or internal, and may have malicious or subjective motives. Individuals may be members of loosely coordinated groups, organizations, and foreign or domestic government agencies. They may be motivated by financial gain, vengeance, ideological views, cyberwarfare, or they may be an intellectual challenge [23]. There are two basic scenarios that an attacker might exploit: operational and technological. Operational scenarios are described as attacks (multistaged or targeted) that occur over a given timeframe and include both technical and operational components throughout the detect–mitigate–respond stages of an attack scenario. These are often more complex and follow a low-and-slow attack technique that relies heavily on human input and intuition in the strategy. Technical (proactive) scenarios are mostly concerned with network anomalies and disruption.

In summary, an adversary targeting the CCAV and its ecosystem should have the capability to study, practice, and instrument an attack by reverse engineering, modifying, replacing, and remotely injecting malicious codes that alter firmware and software pertaining to respective hardware in the cloud-assisted architecture for CAVs [24,25]. The following are the attributes of an adversary that have been considered for this research:

- *Threat Agent:* The adversarial entity that has set its aims on a particular victim.
- *Motivation:* The attacker’s motivations in terms of the benefit he seeks by carrying out the attack.
- *Adversary Capability:* Distinct capability and skills of the adversary.
- *Opportunity:* Indicates the resources and opportunities that are required for the group of threat agents to identify and exploit the vulnerability.
- *Threat:* A cyberattack is a hostile act intended to harm, steal, or disrupt digital assets. A cyber threat is an attempt to obtain unauthorized access to, damage, disrupt, or steal an information technology asset, computer network, intellectual property, or other sensitive data.
- *Tactic:* Tactics are the most abstract level of the MITRE ATT&CK technique. They are the tactical objectives pursued by an adversary during an attack.
- *Technique:* The ATT&CK model’s tactics outline an adversary’s goal. Each tactic category has an endless variety of techniques and subtechniques.

## 5. Research Methodology

Our research methodology uses STRIDE-DREAD to analyze impacted trust domains. We first establish the DFDs for CCAVs, cloud, and edge cloud to enable a more in-depth analysis of threats. The DFDs are described in Table A1, Table A3 and Table A5, which detail each trust domain process, threat entries, and its impact. Our data came from research into real-life incidents (R) and threats detailed in the literature (L), as discussed in [8].

With this knowledge of trust domains and threats, we performed the STRIDE-DREAD analysis on each trust domain. We determined the potential impact of threats found through R and L and classified them as high, medium, and low risks. To understand the risk distribution of threats on trust domains, we demonstrated our results using pie charts. Then, we demonstrated compromised security requirements and assessed the risks by creating Sankey charts.

The outcome of our STRIDE/DREAD analysis for *L* and *R* was mapped. This mapping facilitated the development of an attack tree, which indicates the attack pathways to compromise a CCAV system. After systematically assessing the threats to CCAV security, we created a defense taxonomy that summarizes the countermeasures against attack mechanisms, which were identified in [8]. This helped us in better understanding the validity of overlaps between *L* and *R*, while mapping with the hardware and software measures. Finally, we suggested immediate countermeasures.

Recognizing the importance of comprehensive methods in ensuring the validity and reliability of our findings, certain improvements can be made in our methodology. Firstly, our data collection procedures will be iteratively reassessed and updated to further improve the representativeness of our sample. In the interest of minimizing bias further, we examine and control for potential confounding variables and refine our experimental design to further reduce errors and increase accuracy. Furthermore, our methodology will benefit from integrating Advanced Persistent Threats (APT) and the use of Machine Learning (ML) and Deep Learning (DL) to perform threat ranking to increase its capability to assess qualitative and quantitative threat-related information in a single set of processes. These improvements are aimed at significantly strengthening the longevity and credibility of our study as part of future work.

## 6. Results

This section presents the findings derived from the CCAV model in detail. The respective findings are detailed in (a) System Architecture, (b) STRIDE/DREAD, (c) Impacts on Trust Domains, (d) Impacts on Security Requirements, (e) Risk-Based Classification of Trust Domains with Attack Mechanisms.

### 6.1. System Architecture

**Figure 2:**
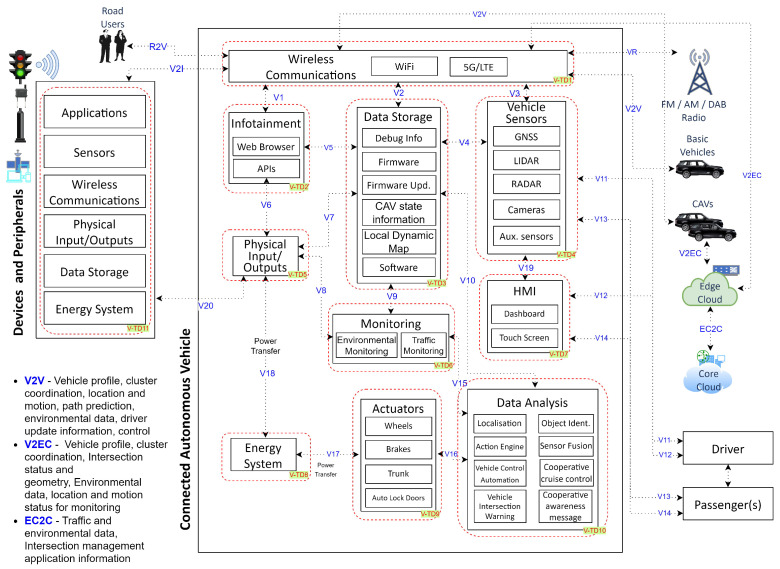
Cloud-Assisted Connected and Autonomous Vehicles Architecture.

Figure 2 illustrates the operational aspects of CCAVs with trust domains, which were identified in [8]. It broadly comprises Devices and Peripherals, CAV systems, Cloud and Edge Cloud, Radio FM/AM/DAB, and Drivers and Passengers. The system comprises subcomponents that interact among themselves. It captures both the internal and external connections of components of CCAVs, with a focus on the key assets utilized in conjunction with infrastructure-enhanced cooperative cruise control application [11]. To achieve this, the study adapts reference architecture originally proposed by [5], customizing it for a three-tier architecture that suits the specific requirements of CCAVs. This is performed to simplify the complex network, identify trust domains, and analyze potential threats.

The DFD shows how CCAVs operate by communicating internally with Electronic Control Units (ECUs) to actuate brakes, steering, and the infotainment system. Controller Area Network (CAN), FlexRay, Media Oriented System Transport (MOST), and Local Interconnect Network (LIN) communication protocols link different ECUs to endpoints such as Tyre Pressure Monitoring Systems (TPMS), infotainment systems, cameras, LIDAR, RADAR, brakes, and actuators [26]. Details of the data flow and the 11 trust domains are described in Table A1.

**Figure 3:**
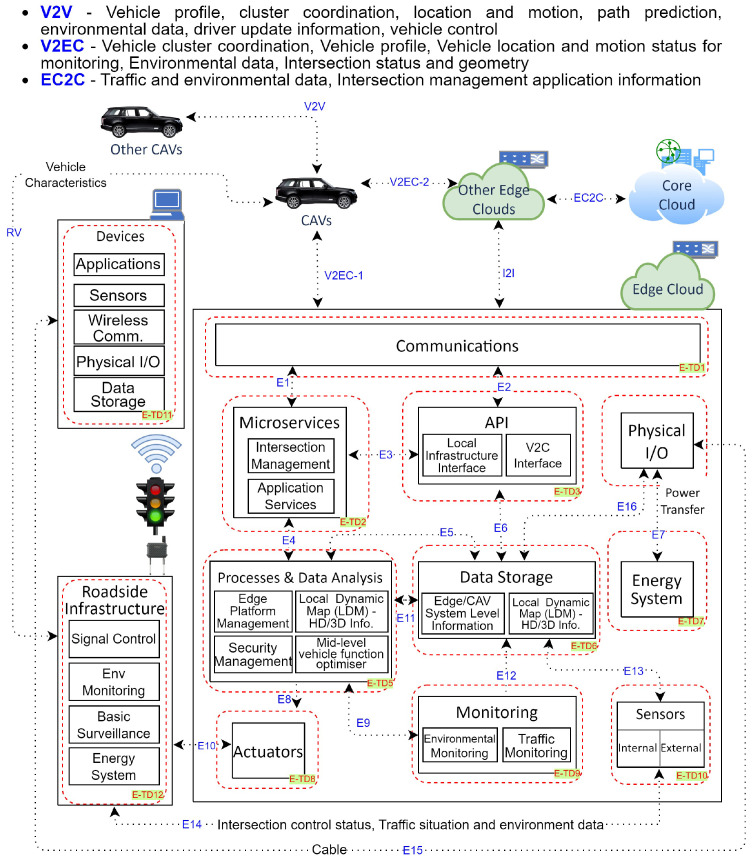
Cloud-Assisted Connected and Autonomous Vehicles—Edge cloud architecture.

**Figure 4:**
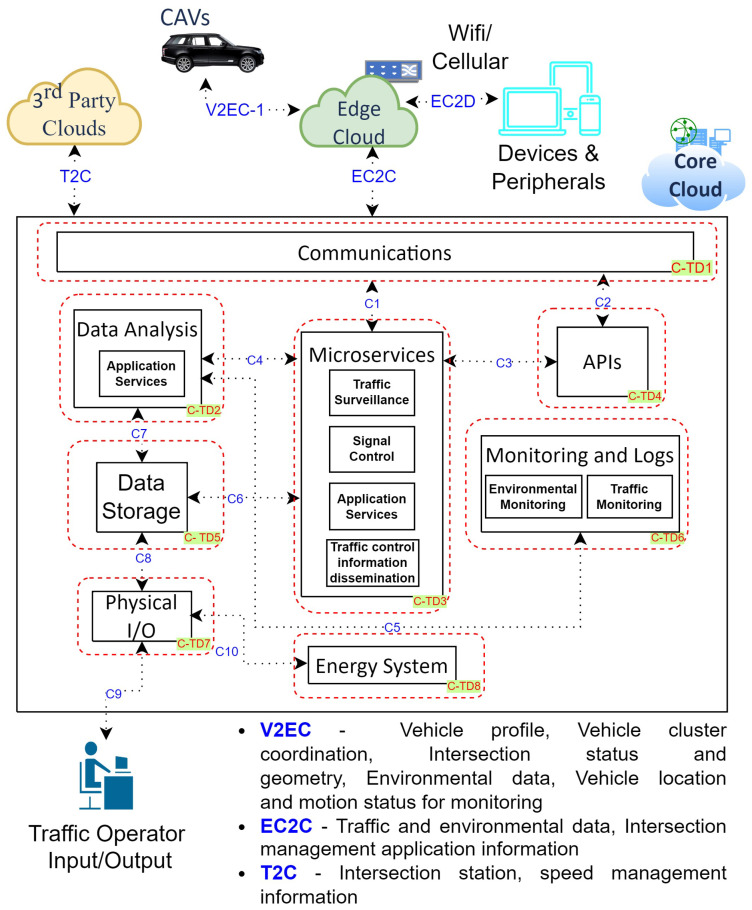
Cloud-Assisted Connected and Autonomous Vehicles—Cloud architecture.

Similarly, Figure 3 and Figure 4 demonstrate the functions of edge cloud and cloud for CCAVs, which aids threat examination [26]. The systems comprise 12 trust domains for edge cloud and 8 trust domains for cloud. These include Devices and Peripherals, as well as Roadside Infrastructures for Edge Cloud and Cloud. Table A2 and Table A3 present a detailed overview of the data flow paths and processes within trust domains, including descriptions of these domains and the potential threat entry/exit points for both the cloud and edge cloud.

### 6.2. STRIDE-DREAD

**Figure A1:**
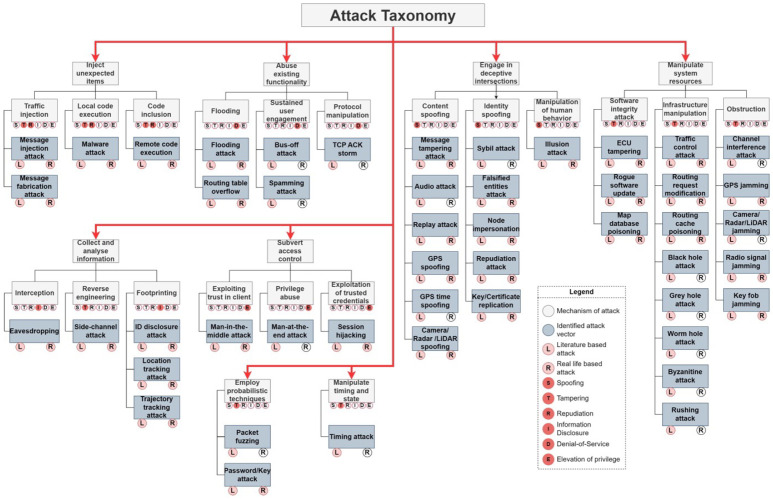
Mapping of documented threats, vulnerabilities, and attacks from the literature and real-world attacks against STRIDE onto a modified attack taxonomy based on CAPEC-1000 attack mechanisms [8,154].

Our evaluation has measured the severity of risks linked to the detected threats using the DREAD methodology on a 5-point scale, as shown in Equation (1). Threats with an average DREAD value of 3.8 or more are classified as High-risk, those between 2.8 and 3.8 are considered Medium-risk, and those scoring less than 2.8 are deemed Low-risk threats. After identifying the threats from the literature review and applying STRIDE methodology, a further investigation of 63 real-life attacks between the year 2015 and the end of 2022 on CAVs was carried out and detailed in [8]. These threats assist in validating the literature review threats. Table A4 and Table A5 summarize and list the types of CCAV attacks that have been studied, predicted, and conducted on systems that compromise confidentiality, integrity, and availability [25,27,28,29,30,31,32,33,34]. The following observations, shown in Table 3, were collected throughout the TARA phases from the threats initially exposed to the CCAV system. Further details on the threats and their classification based on identified attack vectors can be referred to in Figure A1.

### 6.3. Impact on Trust Domains

**Figure 5:**
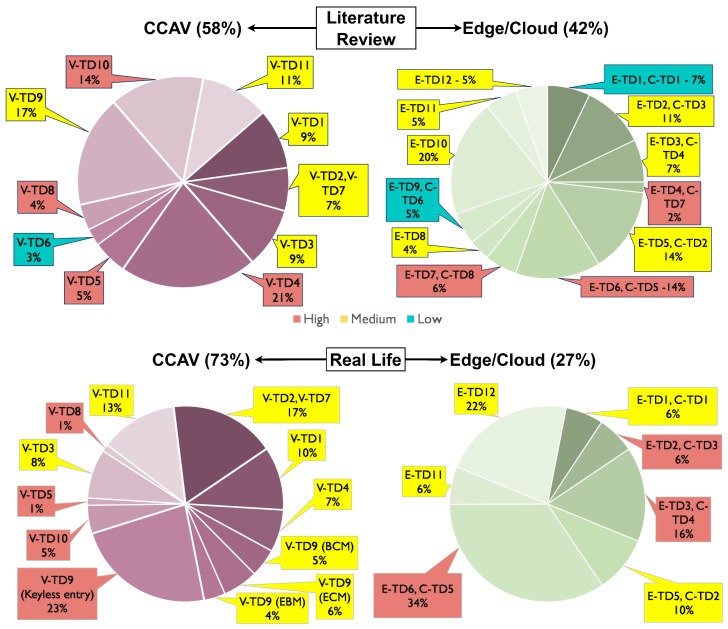
Literature review and Real-Life threat—analysis.

This study concentrates solely on the threats sourced from the Literature and Real Life. Upon analysis, we found that a single identified threat could have the potential to compromise multiple trust domains. The averaged values of risk levels on the impacted trust domains due to the identified threats from both the Literature review and Real Life are represented in the pie chart shown in Figure 5.

Within the CCAV system, 44% of detected Literature threats are classified as high risk, primarily affecting the V-TD4 Vehicle’s Sensors, V-TD5 Physical Input/Output, V-TD8 Energy System, and V-TD10 Data Analysis. Similarly, 30% of the identified Real-Life threats are considered high-risk and have significant implications on V-TD5 Physical Input/Output, V-TD8 Energy System, V-TD9 Keyless Entry System, and V-TD10 Data Analysis trust domains. This study underscores the reality that, despite their identification through research, these high-risk vulnerabilities continue to be exploited in real-world scenarios, presenting a pressing problem that requires immediate attention.

In the context of edge/cloud systems, 22% of the Literature threats are identified as high-risk, predominantly affecting E-TD4, C-TD7 Physical Input/Output, E-TD6, C-TD5 Data Storage, and the E-TD7, C-TD8 Energy System. On the contrary, Real-Life threats suggest that 56% of attacks significantly impact E-TD2, C-TD3 Microservices, E-TD3, C-TD4 APIs, and E-TD6, C-TD5 Data Storage, with Data Storage alone accounting for 34%. These findings illustrate the discrepancies in the affected trust domains, with Data Storage emerging as the most frequently targeted. This vulnerability could stem from the escalating competition for data and antagonistic interests targeting stored data. Another contributing factor might be the relatively slow commercial adoption of Edge/Cloud technologies for CCAV applications, hinting at the possibility of future threats that could lead to operational failures in the system.

When it comes to Literature threats in CCAVs, a small percentage of 3% carries a low risk to the V-TD6 Monitoring module, whereas 12% displays low risks for the E-TD1, C-TD1 Wireless Communication Modules, as well as the E-TD9, C-TD6 Actuator modules. However, no low-risk Real-Life threats have been identified within the CCAV systems. One potential explanation for these observations is that these types of attacks might not be widely reported in mainstream media due to strategic decisions made by organizations to mitigate potential harm to their reputation.

The residual 53% of the Literature threats on CCAVs and 66% on Edge/Cloud collectively constitute the medium-risk category. Intriguingly, our analysis did not find any Real-Life threat reports related to V-TD8 Energy Systems for CCAV or E-TD4, C-TD7 Physical Input/Output, E-TD7, C-TD8 Energy Systems, E-TD8 Actuators, E-TD9, C-TD6 Monitoring, and E-TD10 Sensors for Edge/Cloud trust domains. The remaining Real-Life threats across other trust domains exhibit medium risks, accounting for 70% for CCAVs and 44% for Edge/Cloud systems.

The results highlight that a substantial portion of both the Literature and Real-Life threats on CCAVs and Edge/Cloud systems fall within the medium risk category. Furthermore, the results suggest that while theoretical models predict vulnerabilities in these areas, they may not yet have been exploited or reported in real-life scenarios. Overall, these insights emphasize the need for ongoing research and proactive threat management strategies, particularly in high-risk areas and emerging technologies. They also highlight the value of comprehensive threat reporting, without which our understanding of vulnerabilities in CCAV systems would remain nonexistent.

### 6.4. Impact on Security Requirements

**Figure 6:**
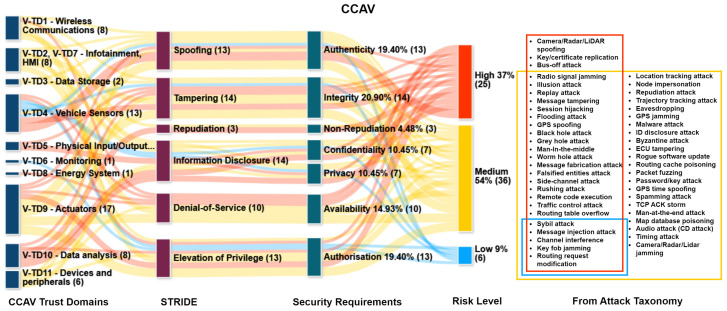
CCAV Sankey diagram describing respective trust domains, STRIDE, compromised security requirements, and criticality of the risk.

**Figure 7:**
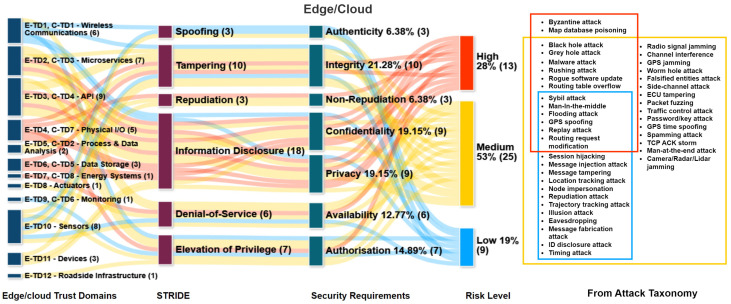
Edge and core cloud Sankey diagram describing respective trust domains, STRIDE, compromised security requirements, and criticality of the risk.

Figure 6 and Figure 7 present an examination of trust domains, STRIDE threats, security requirements, and risk severity related to CCAVs and Edge/Cloud systems. This study also recognizes privacy as a key requirement, as represented in the Sankey diagrams. This is an evolving area of research. Historically, privacy and security were perceived as mutually exclusive. These distinctions were drawn during the design and operation of systems that aimed to provide lawful data access and modifications. Decisions regarding information access and alterations in a challenging and intricate environment like CCAVs have always been distinct from considerations of security, legal compliance, and regulations. Consequently, the idea that privacy and security are not mutually exclusive has gained acceptance.

A common contention made against data privacy is that it cannot be achieved without ensuring security. However, the reverse—that security implies privacy—may not always hold true. Many people tend to equate a technology’s effectiveness with its ability to provide privacy and security. Nevertheless, to develop new privacy and security solutions, further research is required to understand the discrepancies and synergies between these two disciplines. Consequently, this could assist in identifying elements that ensure privacy principles compatible with comprehensive CCAV security [35].

The data derived from Figure 6 indicate that CCAVs are subject to medium risks (54%), high risks (37%), and low risks (9%). It is noteworthy that there are no low-risk instances for availability and nonrepudiation. In relation to security requirements, this study reveals that breaches of confidentiality account for 10.5% of compromises, integrity breaches for 20.9%, and availability breaches for 14.9%. Additionally, breaches of authenticity and authorization each make up 19.4% of compromises. Overall, privacy breaches represent 10.4% of the total security compromise. When it comes to the classification of threats, 26 are deemed high risk, with three of them (sensor spoofing, key/certificate replication, and Bus-off) presenting exclusively high risks. The other 23 threats also contribute to medium risks, and an additional 22 threats pose only medium-level risks. Interestingly, five of the threats classified as low risk also pose both high and medium risks to the CCAV system.

The data derived from Figure 7 for Edge/Cloud systems reveal risk patterns akin to those observed in CCAVs. Medium-level risks are the most prevalent at 53%, trailed by high-level risks at 28% and low-level risks at 19%. In terms of security requirements, the study finds that breaches of confidentiality, integrity, and availability account for 53.2% of the total compromises in Edge/Cloud systems, compared with 46.3% in CCAVs. Breaches of authenticity and authorization together contribute to 21.2% of security compromises in Edge/Cloud systems, as opposed to 38.8% in CCAVs. Notably, privacy breaches occur approximately 9% more frequently in Edge/Cloud systems compared with CCAV systems. When evaluating threats, 14 are identified as high risks in Edge/Cloud systems. Two of these threats, specifically Byzantine attack and Map database poisoning, present only high-level risks. Six threats span both medium and high-risk categories, while the remaining six impact all three risk levels. Moreover, 12 out of the remaining low-level risk threats also carry medium-level risks, and an additional 15 threats pose only medium-level risks.

In summary, the findings unveil a complex and dynamic security landscape, marked by a variety of emerging threats and risks. Addressing these challenges will necessitate comprehensive, agile, and multidimensional security strategies and frameworks that are not only capable of responding to the current threat environment but also equipped to anticipate and prepare for future developments. This implies that there is a need for continuous monitoring mechanisms with advanced predictive analytics to counteract emerging threats, which may involve machine learning and artificial intelligence. Additionally, the study highlights an imperative need for a paradigm shift in understanding and ensuring data privacy, which is often overlooked. Effective strategies would recognize the intricate relationship between security and privacy.

## 7. Analysis and Discussion

In this study, we executed a TARA methodology utilizing the STRIDE model to systematically capture threats and quantify risks. In addition, this research has quantified risks with the DREAD model. Limitations inherent to this approach for identifying threats in hardware and software components are discussed in this section. This technique assists in distinguishing the impact of current controls, thus enabling the identification of both strengths and weaknesses within the CCAV system. This paper also used mature CCAV reference architectures with assets identified systematically in [8]. This further enables us to learn attack pathways by constructing detailed attack trees. From this discussion, key countermeasures are developed in alignment with existing standards. This progression is crucial in guiding further research in this domain.

### 7.1. Threats and Risks

Threat Identification, Threat Distribution, and Refinement of Threat Understanding require further analysis to gain insight into the complex landscape of risks.

- *Threat Identification and Distribution*: This study identifies a variety of threats with some, such as Byzantine attacks and Map database poisoning, exclusively posing ‘Critical’-level risks, while others affect multiple risk levels. This complexity and diversity of threats necessitate ongoing threat intelligence and assessment.
- *Risk Identification and Distribution*: This research infers high-level risks as ‘Critical’, medium-level risks as ‘Warning’, and low-level risks as ‘Caution’. The findings reveal a significant proportion of ‘Warning’-level risks in both CCAVs and Edge/Cloud systems, constituting 54% and 53%, respectively. While the presence of ‘Critical’-level risks is indeed concerning, the dominance of ‘Warning’-level risks highlights the ongoing security challenge that needs to be managed with a multilayered security strategy. The overlapping impact of threats across all risk categories (’Critical’, ‘Warning’, and ‘Caution’) adds a layer of complexity to the system’s security, emphasizing the need to strategise proactive measures. A threat that is considered ‘Caution’ or low-risk in one scenario might contribute to a ‘Warning’ or ‘Critical’-risk situation in another, depending on the overall security context.
- *Refinement of Threat Understanding*: Through the use of attack trees, mapping potential attack routes from data flow diagrams, and referencing architecture, our comprehension of potential threats and their vectors would be improved. However, there seems to be an obscurity in the methodology to create security solutions for both hardware and software components, indicating a need for further research and development in this area.

### 7.2. Security Requirements

This subsection delves into the analysis concerning the potential breaches of Confidentiality, Integrity, Availability, Authenticity, Authorization, and Privacy highlighted in the Sankey diagram.

- *Confidentiality, Integrity, and Availability*: Often referred to as the CIA triad, they are revealed as the most frequently compromised security requirements, accounting for a large portion of the total security compromises in Edge/Cloud systems (53.2%) and CCAVs (46.3%). This underscores a fundamental vulnerability in the core security architecture of these systems and the importance of strategic measures to protect the CIA triad.
- *Authenticity and Authorization*: Authenticity and authorization compromises form a smaller but significant proportion of security compromises. There is a stark difference between Edge/Cloud systems (21.2%) and CCAVs (38.8%), highlighting the unique security challenges of each system. This difference could be indicative of more complex user interaction models or greater reliance on trusted access controls in CCAVs.
- *Privacy*: Interestingly, privacy compromises occur 9% more frequently in Edge/Cloud systems compared with CCAVs, likely reflecting the shifting threat landscape driven by the value and volume of data processed by these systems. This also emphasizes the growing challenges posed by stringent data privacy regulations and the need for proactive privacy-by-design approaches.

### 7.3. TARA Limitations

There are several limitations of TARA when applied to CCAVs. Firstly, these methods cannot precisely represent adversarial behavior, especially in targeted or multistage attacks that exploit physical components as attack agents. The existing TARA methodologies struggle to capture the complexity of such attacks, which impairs their effectiveness in safeguarding CCAVs.

It is understandable that traditional approaches like STRIDE and DREAD would face scrutiny for their efficacy. These methods may not fully accommodate the unique security challenges presented by CCAV technology, which is rapidly evolving. Consequently, the need for more robust and comprehensive analysis techniques is necessary to ensure the security of CCAVs against emerging security threats.

A significant gap exists in the systematic security analysis of CCAVs. The focus in the field is primarily on analyzing isolated systems, disregarding the intricate interplay between hardware and software components. This approach fails to capture the vulnerabilities and potential effects that may arise from the complex interactions within a dynamic CCAV ecosystem.

Accurately characterizing the degree to which a CCAV system conforms to security requirements presents a challenge for researchers because it is difficult to define, verify, and validate conformance. In addition to this, there is a need to account for various assumptions about system performance. As a result, security requirements that are poorly defined can lead to insufficient protection against potential threats.

Despite these limitations, protecting CCAVs from identified threats is imperative. To address these challenges, it is essential to develop advanced countermeasures and analysis techniques that protect against the exploitation of CCAV vulnerabilities. The TARA methodology has been instrumental in understanding the necessity for the development and application of countermeasures on both hardware and software components of the systems. To improve our ability to recommend countermeasures, we map attack pathways and construct an attack tree in this section to learn how a CCAV could systematically be compromised. Following this, we develop a defense taxonomy that provides a step toward achieving this objective for subsequent research in the next section.

### 7.4. Attack Tree

**Figure 8:**
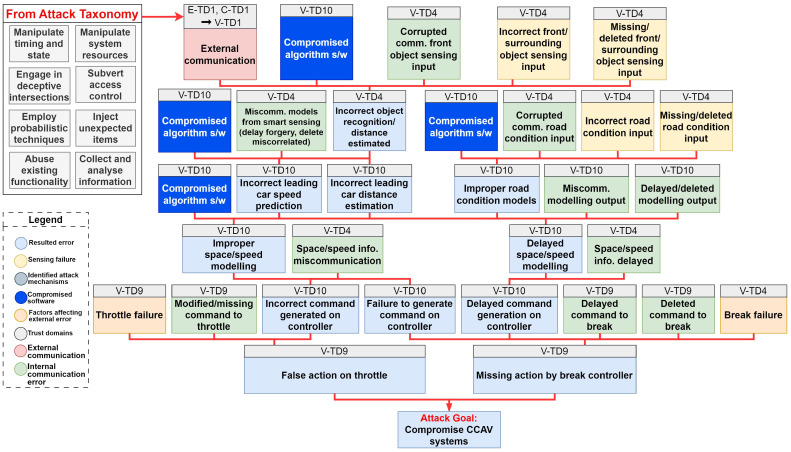
Attack tree capturing attacks from attack taxonomy that illustrates impacts on the generalised CCAV scenario. Further information on Attack taxonomy can be obtained from [8].

The attack tree, illustrated in Figure 8, is a graphical representation of the potential security threats in a generalized CCAV scenario, with the primary source of these vulnerabilities arising from external communications within the Vehicle-to-Everything (V2X) systems. It is crucial to note that the attack tree highlights the adverse consequences of a compromised CCAV system, including the potential for collisions, thereby emphasizing the importance of ensuring the security of these systems. The attack tree validates that there are several attack vectors that show a vehicle is vulnerable through wireless channels or via the onboard system [36,37].

In summary, the findings, taxonomy, and attack tree show that CCAVs are vulnerable. To exploit a CCAV, an adversary may employ a series of attack techniques and varied tactics. The intricacies of these attack mechanisms, which range from external to internal communications, are visually represented in the attack tree shown in Figure 8. This diagram captures potential attacks that could be initiated from vehicle-to-vehicle (V2V) interfaces or from the edge cloud towards CCAVs. Such a visual representation aids in comprehending the multifaceted nature and complexity of potential attacks that could compromise the security requirements of safety-critical CCAVs by impacting specific trust domains.

As seen in Table A5, it is critical to address these issues through strong and efficient security mechanisms such as anomaly detection, tamper-resistant hardware systems, secure software development practices, and the secure segmentation of onboard vehicular networks, which is explored in the following section.

## 8. Countermeasures

Stringent measures can be employed to protect CCAV systems and mitigate potential risks, even in the face of unpredictable and complex threats. Our methodology identified numerous threats to both the hardware and software components of CCAV systems. While providing a high-level understanding of threats, our study focused on analyzing threats at the component level and acknowledged the challenges associated with this approach. To achieve this goal, we mapped attack mechanisms to the hardware and software components of each trust domain within a CCAV system using an Attack Tree (see Section 7). In this section, we propose a classification of hardware and software components to identify attack mechanisms and impacted trust domains. Based on these mechanisms, a defense taxonomy was developed. Drawing insights from the literature, ongoing research, and real-world implementations, we proposed key countermeasures by identifying the impacted trust domains and analyzed them.

### 8.1. Classification of Trust Domains with Attack Mechanisms

The TARA has enhanced our understanding of the need for tailored countermeasures for each hardware and software component. However, there is a notable obscurity in the approach to devising security solutions for both hardware and software. To pinpoint solutions, we categorized various attacks and grouped them according to their attack mechanisms, which were clustered from the attack taxonomy illustrated in [8]. These were then correlated with the affected trust domains, granting us the insight that countermeasures can potentially be suggested based on the classification of attack mechanisms.

**Figure 9:**
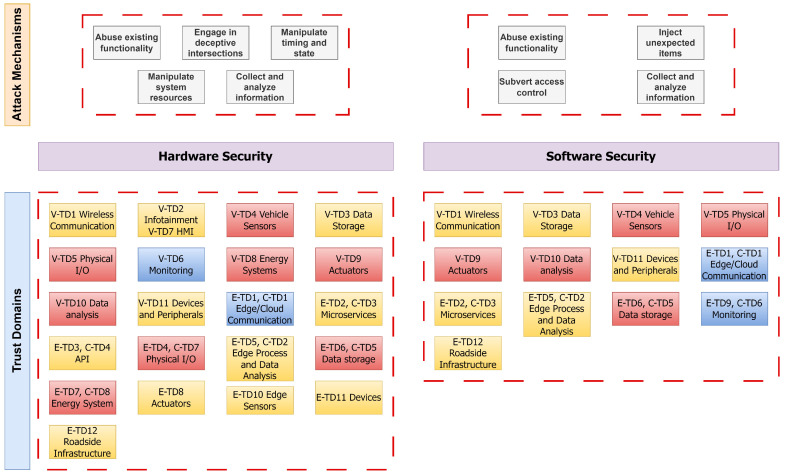
Mapping trust domains to attack mechanism (vectors).

From Figure 9, we can derive that every identified trust domain is encompassed within the security of hardware and software components. Regarding hardware attacks, a total of 28 trust domains are affected: all 11 trust domains under the CCAV are impacted, alongside 17 of the edge and cloud trust domains, excluding the monitoring systems. Of these, 11 trust domains are identified as high risk, 14 are considered medium risk, and 3 are considered low risk based on our previous evaluations. In terms of software security, 18 trust domains are impacted: 7 are related to CCAV and the remaining 11 pertain to edge and cloud systems. Among these, six trust domains are classified as high-risk, seven as medium-risk, and four as low-risk, again, following our preceding analysis

### 8.2. Defense Taxonomy Based on Attack Mechanisms

**Figure 10:**
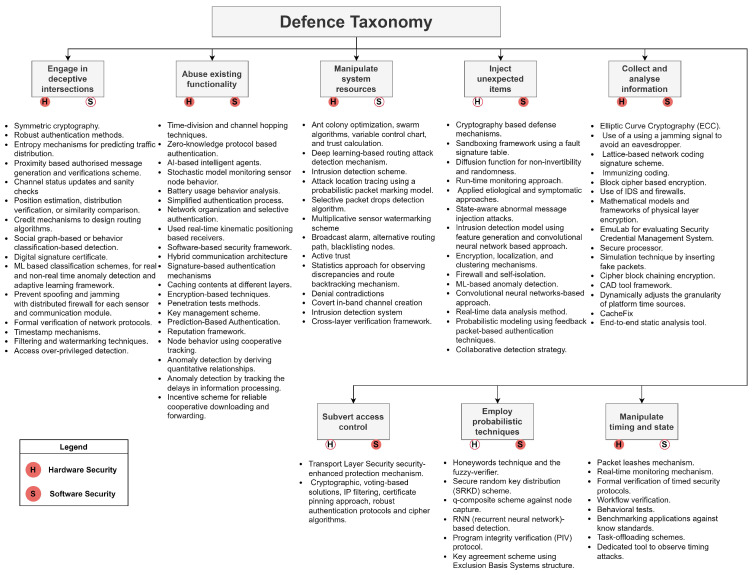
Defence taxonomy for CCAV attack mechanisms.

**Figure A2:**
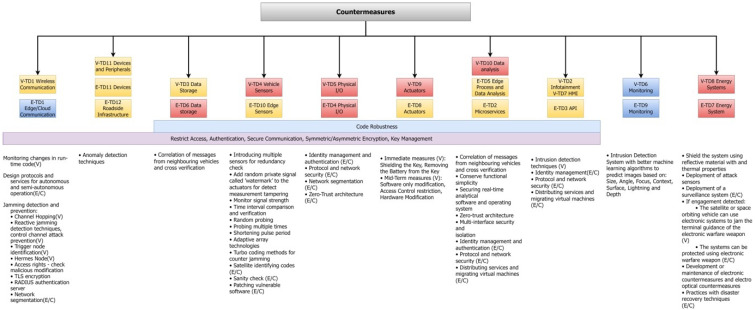
Detailed defense taxonomy for CCAV attack mechanisms.

Figure 9 underscores the need for a multifaceted strategy to safeguard trust domains in CCAV systems, entailing a blend of diverse yet interrelated countermeasures tailored to specific attack vectors. Building a defense taxonomy is a two-step process: first, mapping trust domains and then curating the taxonomy using identified attack mechanisms. An extensive list of countermeasures from Table A4 and Table A5, labeled as Hardware Security *(H)* and Software Software *(S)*, is aligned to these attack mechanisms, as depicted in Figure 10. While this figure depicts a comprehensive list, the subsequent section discusses key immediate countermeasures. Further details on countermeasures for each trust domain can be referred to in Figure A2.

### 8.3. Key CCAV Countermeasures

It is important to consider key security practices when adopting new countermeasures for the secure development lifecycle of a vehicle. Miller and Valasek [26] assert that vehicles should be designed with safety prioritized and recommend defensive technologies, such as an Intrusion Detection System (IDS), to prevent attacks on the CAN bus. To detect an attack, histogram analysis of diagnostic packets during the CAN bus operation could study the repetitive nature of system messages and detect anomalies that indicate a deviation from normal operation. Moreover, the National Highway Traffic Safety Administration [38] has proposed a layered approach to harden vehicle electronics. The approach employs preventive measures by isolating safety-critical and identification systems, utilizing intrusion detection and real-time threat response, and regularly assessing comprehensive system solutions. These solutions are shared collaboratively, incorporating data from past security threats between partners and organizations.

Bariah et al. [30] discussed security mechanisms including PKI, ID-based cryptosystem, and situation-based mechanisms. PKI ensures data authentication and nonrepudiation but lacks consistent location privacy. ID-based mechanisms use user/vehicle information for verification and offer pseudonym generation for privacy but incur additional overhead. The authors found that ID-based mechanisms outperform PKI in terms of time, bandwidth, and storage. Situation-based modeling helps monitor, analyze, and ensure security but requires high computational resources for spontaneous sensing and model extraction.

Amoozadeh et al. [33] proposed countermeasures such as local plausibility checks and infusing information from external devices. However, this approach introduces additional risks and attack vectors through smartphones and wearables, which could be used to inject malicious packets into a vehicle. Limitations include unreliable smartphone processing power, trustworthiness of third-party device applications, and confidence in verifying data with onboard systems. The authors also suggested using voting as a countermeasure, where vehicles track each other’s behavior to identify anomalies. However, further research is needed to improve computational and communication overheads for voting mechanisms [33].

#### 8.3.1. Hardware Security Module

To ensure the security and trustworthiness, of CCAVs, robust hardware-based security measures are crucial. Hardware Security Modules (HSMs), or Trusted Platform Modules (TPMs), are effective in authenticating and protecting onboard systems. These specialized devices securely store and process sensitive data, preventing tampering, unauthorized access, and malware attacks. They safeguard the integrity and confidentiality of vehicle location data, sensor data, and communication keys. However, research and development have progressed privately and as such, public knowledge and understanding are limited. Proprietary solutions in the automotive industry may suppress innovation and result in legal challenges. Collaboration and liability policies among technology companies, automotive OEMs, and governments are essential for secure data logging and forensic analysis during cyber incidents [24,27,39,40].

#### 8.3.2. Cryptographic Solutions—Encryption and Authentication

To enhance security in CCAVs and associated infrastructure systems, various encryption and authentication mechanisms have been recommended. However, implementing these techniques poses challenges due to resource constraints and limited computational power in vehicles. The complexity is exacerbated when vehicles are built containing varying hardware and software from different manufacturers. Therefore, relying on a single static security mechanism is insufficient, and multiple security mechanisms are needed for onboard and remote systems. Recent research focuses on short-term certificates, pseudonyms, and efficient revocation lists using Trusted Authorities (TA) and PKI with support from RSUs [41,42].

#### 8.3.3. Software Updates

To address security vulnerabilities in manufactured vehicles, Over-the-Air (OTA) software updates from edge or cloud have been proposed as a solution for upgrading and fixing software vulnerabilities. While OTA updates can address onboard vulnerabilities, there is a risk of executing remote codes through malicious OTA updates. If not implemented securely, this can enable adversaries to inject malware and execute remote code [43]. Research on the probability and impact of various vulnerabilities on compromised software, hardware, and sensors in vehicles has been limited [44]. Resilience is crucial for safety-critical CCAV systems, and implementing redundant systems can enhance resilience. However, the characteristics of redundant system technologies, such as communication and perception sensors, are still unclear. Research on reliable and resilient software systems is significant but currently limited.

#### 8.3.4. Anomaly Detection Mechanisms

Attention to cloud-based security solutions for securing CCAVs has been growing over time. Companies like HERE, Ericsson, IBM, CloudCar, GM On-Star, and Amazon AWS have previously discussed centralized cloud architectures [45]. Amazon AWS and Ericsson have proposed connected vehicle cloud systems that give anomaly detection in the cloud infrastructure greater weight in detecting malicious data packets using machine learning algorithms. Detected anomalies are stored in a local database and shared with other vehicles for notification [43,46]. Academic research suggests using vehicle trajectory, data clustering, and entropy-based attack detection to enable the systematic surveillance of anomalies among vehicles in a CCAV’s three-tier architecture [47,48,49].

### 8.4. Analysis of Countermeasures

As seen in Figure 9, it is apparent that the identified trust domains encompass both hardware and software security elements. The risk levels within this cluster are diverse for both hardware and software security. This reveals the specific vulnerabilities of each element and emphasizes the necessity of specialized countermeasures for respective component security. This suggests that a ‘one-size-fits-all’ approach may not be sufficient in developing robust and effective security measures.

The above points indicate the importance of a holistic, component-level security approach. Both hardware and software elements need to be shielded against potential attacks. The defensive measures should be developed based on a comprehensive understanding of the system, its components, and their vulnerabilities. This understanding, coupled with threat and risk assessments, should enable organizations to establish robust countermeasures capable of safeguarding their systems against High—’Critical’, Medium—’Warning’, and Low—’Caution’-level risks.

Countermeasures have been grouped based on their similarities among trust domains in CCAVs, edge cloud, and cloud, such as V-TD1, E-TD1 and C-TD1 (Wireless Communication Modules). Upon analysis, this research found various countermeasures that can protect multiple trust domains in CCAV systems against threats ranked between *low-* and *medium*-level risks and those with *medium-* to *high*-level risks:

- *Low- to Medium-Level Risks*: It is found that wireless communications can be protected from jamming attacks using preventive measures such as reactive jamming detection techniques, control channel attack prevention, trigger node identification, Hermes node, checking access rights, and TLS encryption, as well as anomaly detection techniques. This research recommends intrusion detection systems with optimized machine learning algorithms to safeguard devices, peripherals, and RSUs against adversarial attacks.
- *Medium- to High-Level Risks*: It is found that various countermeasures, such as identity management and authentication, protocol and network security, network segmentation, and Zero-Trust architecture, for protecting trust domains such as Physical Input/Output, Data Analysis, Microservice, and Sensors. In addition, the correlation of messages from neighboring vehicles and cross-verification can protect data storage, analysis, and microservices.

In summary, the authors discussed various security mechanisms for monitoring onboard network traffic in CCAVs. However, solutions addressing dynamic security features are limited, especially considering the low latency requirements of safety-critical applications in vehicles. Key areas that need attention include managing communication latency due to security overhead, minimizing message routing delays, and finding solutions for vehicle dependency on the cloud when infected with malware. There is a significant research gap in utilizing machine learning and artificial intelligence methods to predict threats and develop security mechanisms based on node behaviors; however, due to the unexplainable nature of artificial intelligence in making decisions within CCAV systems, the field remains a grave concern to passenger safety. Although there is a lack of clarity in the methodology for developing comprehensive security solutions for hardware and software. An effort was made to identify effective solutions by categorizing attacks based on attack mechanisms.

## 9. Conclusions

In conclusion, this paper presents a detailed study through a successful examination of the efficacy of STRIDE/DREAD in capturing security requirements and threats from a system-centric perspective in CCAVs. The objectives were successfully achieved through the formulation and analysis of CCAV architectures. A systematic threat analysis and risk assessment were conducted to evaluate the impact on trust domains and security requirements. Based on the findings, countermeasures were analyzed and suggested, utilizing a defense taxonomy that mapped the hardware and software components of CCAV systems.

**Figure A3:**
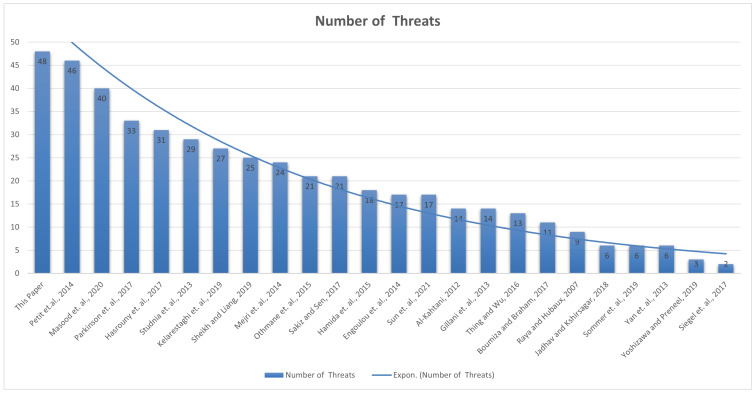
Number of identified threats from the taxonomy against other papers in the literature [3,23,25,27,29,34,39,40,50,51,52,53,54,55,56,57,58,59,60,61,62,63,64], adapted from [8].

This research differentiates itself from other works in the field of CCAVs, as indicated in Table 4 and Figure A3. It systematically discusses a larger number of threats in greater depth while quantifying risks, contributing to the development of novel defense taxonomy. It delves into system architecture, threats, and countermeasures to understand and protect the respective hardware-software assets, which are critical for overall system safety. By abstracting the hardware and software components, the study investigates the security requirements of the CCAV system and its components in their ecosystem. This approach addresses a significant gap in the existing literature, particularly in the understanding of hardware–software interaction in the CCAV ecosystem, which is an open area for scientific inquiry. Future research will likely provide a better understanding of this limitation.

This paper is comprehensive because of its expansive coverage of critical topics, notably including threat analysis, risk assessment, and attack tree analysis. These elements, essential for a thorough understanding of security threats in vehicular systems, are frequently either overlooked or only partially addressed in other studies. This comprehensive inclusion sets this paper apart from other works in Table 4, like those by [39,50], which primarily concentrate on V2V/V2I communications or in-vehicle networks. Additionally, this paper broadens the scope explored in studies such as [25,29], offering a more holistic perspective on the field.

Methodologically, this paper employs STRIDE-DREAD modeling and a comprehensive literature review to analyze security threats in vehicular systems, a technique not commonly employed in studies in [60,61]. This paper’s rigorous evaluation of existing literature, assessing both the strengths and limitations of prior research, aligns with but extends beyond the analyses in works like [63,64]. Furthermore, the focus on defense taxonomy and countermeasures, particularly for prioritized threats based on risk, marks a significant advancement in research. This focus, coupled with discussions of the TARA applied to detailed reference architectures, highlights this paper’s comprehensive approach to addressing challenges in CCAVs. Such integration of diverse elements builds upon and enhances the foundational research conducted by scholars like [3,34,53,55,58], further solidifying this paper as a pivotal contribution to the field of CCAV security.

The proposed systematic approach for analyzing threats and risks in CCAV offers valuable insights for informed decision making in risk management. It covers important considerations for security and privacy to legal compliance, providing a foundation for future research in this field. This approach benefits stakeholders, security and privacy experts with libraries of vulnerabilities and threats, and engineers for secure coding practices with protocols. By following this approach, organizations can ensure they are in line with the latest industry standards and best practices to consider security and privacy by design.

However, there are some notable limitations to the TARA when used to analyze CCAVs. These include the inability to accurately represent complex adversarial behavior, particularly in targeted or multistage attacks that exploit physical components. Furthermore, STRIDE and DREAD may not be effective in addressing the unique security challenges of CCAV technology, which is quickly advancing. The current analysis techniques often overlook the relationship between hardware and software components and therefore fail to capture vulnerabilities due to their interaction and their potential impact within the CCAV ecosystem. The authors conclude this research with a recommendation that future research may aim to develop and employ alternative modeling techniques to analyze security threats in CCAV systems, better capture intricate vulnerabilities, and inform the latest countermeasures.

## Figures and Tables

**Table 1:** Evaluation of the threat modeling methods (M: Maturity, A: Adaptability, SS: Safety and Security Dependency, H/S: Hardware and Software Threats, D: Documentation).

Method	M	A	SS	H/S	D
Attack Trees [22]	✓	✓		✓	✓
Composite Threat Modeling [17]	✓		✓	✓	✓
OCTAVE [15]	✓				✓
PASTA [16]	✓	✓		✓	✓
STRIDE DREAD [9]	✓	✓	✓	✓	✓

**Table 2:** Adversarial model.

Expertise	Threat Actor	Motivation	Capability	Opportunity	Threat	DREAD
Layman	Solo—Outsider	Personal satisfaction; Passion; Ideology.	Limited	Minimal		Damage Potential —If a threat exploit occurs, evaluate the damage caused 0 = Nothing 2.5 = Individual user data compromised 5 = Complete system or data destruction *Reproducibility*—How easy is it to reproduce the threat exploit? 0 = Very hard or impossible even for administrators/DBAs 2.5 = One or two steps required, may need an authorized user 5 = Just a web browser is enough *Exploitability*—What is needed to exploit this threat? 0 = Advanced programming networking knowledge 2.5 = Malware exists on the Net, or any tolls available 5 = Just a web browser is enough *Affected Users*—How many users are affected? 0 = None 2.5 = Some users, but not all 5 = All users *Discoverability*—How easy is it to discover the threat. 0 = Very hard or impossible; needs source code or admin access 2.5 = Can figure it out by guessing or monitoring network traces 5 = Information is visible in the web browser or address bar or in the form or as a hidden variable
Proficient	Solo—Insider	Financial gain; Discontent	Moderate to High	Internal knowledge	S	
	Group—Ad hoc	Dependant on group purpose: Ideological, financial, political	Limited to Moderate	Limited knowledge and financial	T R	
Expert	Group—Established	Dependant on group purpose: Ideological, financial, political	Limited and Moderate knowledge	Moderate to High	I	
Multiple experts	Organization—Competitor	Corporate espionage; Financial gain; Reputation damage	Moderate to High	Limited and moderate knowledge and financial and contextual	D	
	Organization—Partner	Information gain; Financial gain			E	
Intelligence RD	Nation-State	State rivalry; Geopolitics	High	High knowledge, finance and advance skills and resources		

**Table 3:** The number of threats categorised as Low (Caution-Blue), Medium (Warning-Yellow), or High (Critical-Red).

	Low	Medium	High
**Literature Review Threats**			
CAVs	2	40	34
Edge/Cloud and Cloud	7	37	12
**Real-Life Threats**			
CAVs	3	47	36
Edge/Cloud and Cloud	1	14	17

**Table 4:** Comparison with surveys related to cybersecurity of CCAVs.

Surveys	Reference Architecture	CAV	Edge/ Cloud	V2V/V2I Comm.	In-vehicle Network	Threat Analysis	Risk Assessment	Defense Taxonomy	Attack Tree	Counter Measures
[39]	✗	✗	✗	✓	✗	✗	✗	✓	✗	✓
[25]	✗	✗	✗	✓	✓	✗	✗	✗	✗	✗
[27]	✗	✗	✗	✓	✓	✗	✗	✗	✗	✓
[29]	✗	✗	✗	✓	✗	✗	✗	✓	✗	✓
[50]	✗	✗	✗	✓	✓	✗	✗	✗	✗	✗
[51]	✗	✗	✗	✓	✗	✗	✗	✗	✗	✓
[23,40]	✗	✗	✗	✓	✓	✗	✗	✓	✗	✓
[52]	✗	✗	✗	✓	✓	✗	✗	✗	✗	✓
[53]	✗	✗	✗	✓	✗	✗	✗	✗	✗	✓
[54]	✗	✗	✗	✓	✓	✗	✗	✓	✗	✓
[34,55]	✗	✗	✗	✓	✗	✗	✗	✗	✗	✓
[56]	✗	✓	✗	✗	✗	✗	✗	✗	✗	✗
[57]	✗	✗	✗	✓	✗	✗	✗	✓	✗	✓
[58]	✗	✗	✗	✓	✓	✗	✗	✓	✗	✓
[59]	✗	✗	✗	✓	✓	✓	✗	✓	✗	✓
[3]	✗	✗	✗	✓	✗	✓	✗	✓	✗	✓
[60]	✗	✗	✗	✓	✓	✗	✗	✓	✗	✗
[61]	✗	✗	✗	✗	✓	✓	✗	✓	✗	✓
[62]	✗	✗	✗	✓	✗	✗	✗	✗	✗	✓
[63]	✓	✓	✓	✓	✓	✓	✗	✓	✗	✓
[64]	✗	✗	✗	✓	✗	✓	✓	✓	✗	✓
This Paper	✓	✓	✓	✓	✓	✓	✓	✓	✓	✓

Tick (✓) and cross (✗) symbols are used to denote the presence and absence of topics, respectively.

## Data Availability

The data presented in this study are available on request from the corresponding author, A.T.S. The data are not publicly available due to the confidentiality of the research undertaken.

## Appendix A

**Table A1:** CCAV Reference Architecture—Detailed description of Trust Domains, Data Flow ID, Data Processes, Data Flow Description, Threat Entry/Exit from Figure 2.

CCAV Reference Architecture				
**Trust Domain**	**Data Flow ID**	**Data Process**	**Description**	**Threat Entry/Exit**
V-TD1: Wireless Communication	V2V, V2I, R2V, EC2V, VR, V1, V2, V3, VR	Data Transmission	CAVs communicate with the Edge Cloud, other cars and CAVs, possible technologies linked to road users, infrastructures, and radio stations on a frequent basis, depending on the receiver and transmitter’s position and vicinity. DSRC, 5G, 4G/LTE, and other protocols may be used for sharing data, depending on the application.	Communication Unit (Software and Hardware modules), Antenna, V-TD2, V-TD3, TD4, Radio, EC2V, V2V, V2I
V-TD2: Infotainment	V1, V5, V6	Physical Interaction and Data Transmission	It is a group of hardware and software components installed in automobiles that offer audio and visual entertainment. It began with radios with cassette or CD players and has expanded to include navigation systems, video players, USB and Bluetooth connection, internet, and WiFi. Examples include CarPlay and Android Auto. The internal components (Wireless Communication Module, I/O ports, and data storage) can transmit data to this module	Database (eg. SQL), input ports, Wifi, 5G/LTE, V-TD1, V-TD3, V-TD5
V-TD3: Data Storage	V2, V4, V5, V7, V9, V10	Database Access	Vehicles would need storage for data related to audio, video, maps, firmware and its versions, and vehicle status. These records are partitioned and securely stored.	The vehicle’s sensors, monitoring module, physical input/output, and infotainment system supply these data, V-TD1, V-TD2, V-TD5, V-TD4, V-TD9
V-TD4: Vehicle Sensors	V3, V4, V11, V13, V19	Data Processing	Vehicles are often equipped with a plethora of sensors that monitor the vehicle’s motion dynamics and vehicle system. GNSS, LIDAR, RADAR, and cameras are all important sensors for CAVs. Additionally, sensors such as tire pressure monitoring sensors, light sensors, parking sensors, wheel and vehicle speed sensors, and others are considered in this study.	Driver, passengers, environment, V-TD1, V-TD7
		Physical Interaction	The onboard sensors may be exposed to environment-specific threats,	Environment (e.g., fire, radiation, magnetic waves, thermal elements)
V-TD5: Physical Input/ Outputs	V6, V7, V8, V18, V20	Physical Interaction	This module refers to the physical inputs and outputs on the device, such as the USB port, the onboard diagnostic port (OBD-II), and Type 1-4 battery chargers. It is difficult to exploit these ports since they need physical access.	V-TD2, V-TD3, V-TD6, V-TD11
V-TD6: Monitoring	V7, V8, V9, V15	Data processing	This module is used to describe the vehicle’s monitoring function. Here, the vehicle’s operation is verified against its specifications, its history is verified, and the vehicle’s maintenance is documented and logged. A good example is the black box, which is available commercially.	V-TD5, V-TD3, V-TD5, V-TD10
V-TD7: HMI	V11, V12, V13, V14, V19	Phy. interaction, data processing and trans.	The Human–Machine Interface (HMI) is a collection of hardware and software elements that enables an individual to engage actively with the CAV system. It may be used as a user interface for steering wheels equipped with sophisticated onboard displays.	V-TD4
V-TD8: Energy System	V17, V18	Physical Interaction	The onboard energy system may be vulnerable to environmental challenges. It mainly consists of batteries and a fuel tank (petrol or diesel)	Environment (eg. Fire, Radiation, Magnetic waves, Thermal elements), V-TD8, V-TD9
V-TD9: Actuators	V17, V16	Data Processing	This module discusses components that have the potential to influence the physical environment. This includes adjusting the wheel speed and angle, activating the brakes, air conditioning, and windows, as well as locking the doors and trunk.	V-TD8, V-TD10
		Physical Interaction	Physical components receive their energy unit to interact with the environment	Vehicular Environment
V-TD10: Data Analysis	V10, V15, V16	Data Processing	This module is in charge of conducting an analysis on the data that have been saved. This might be for data localization, object recognition, sensor fusion and analysis, action engine decision-making, vehicle control automation, warning, and basic safety message analysis, as well as vehicular applications.	V-TD8, V-TD10
		Physical Interaction	Physical components receive their energy unit to interact with the environment	Vehicular Environment
V-TD11 Devices and Peripherals	V2I, V20	Data Processing and Physical interaction	Smartphones, Bluetooth devices, laptops, and desktop computers are all examples of devices and peripherals. Admins, users, and operators would use these devices to communicate with CCAVs and devices to use the system. These are additional methods via which an adversary may breach the system. COHDA units are used to represent roadside infrastructure. These devices would be utilized by traffic controllers, CAVs, and other edge devices to carry out ITS-based prompts.	Environment, V2I, V20

**Table A2:** Edge Cloud Reference Architecture—Detailed description of Trust Domains, Data Flow ID, Data Processes, Data Flow Description, Threat Entry/Exit from Figure 3.

Edge Cloud Reference Architecture				
**Trust Domain**	**Data Flow ID**	**Data Process**	**Description**	**Threat Entry/Exit**
E-TD1: Wireless Communication	E1, E2, V2EC-1, I2I,	Data Transmission	The communication module presented here is expected to establish wireless connections with nearby automobiles, cloud technologies, RSI, and other peripheral devices through a cellular network or DSRC. They are also linked through fiber-optic cables to the Wide Area Network (WAN).	Communication Unit (Software and Hardware modules), Antenna, E-TD2, E-TD3, CAVS, and Other Edge Clouds
E-TD3: API	E2, E3, E6	Data transmission and interaction	Application Program Interfaces (APIs) are used by users and software modules to obtain access to a specific service.	E-TD1, E-TD2, E-TD6
E-TD2: Microservices	E1, E3, E4	Data Processing and data transmission	The microservices module is in charge of offering services that are composed of multiple services. They are well-known for providing unique services through facilitating scalability and testing. For example, intersection management.	E-TD1, E-TD3, E-TD5
E-TD4: Physical I/O	E15, E16, E7	Phy. interaction and data trans.	Connection to the edge infrastructure is made possible via the physical IO ports. Physical security mechanisms should be used to protect these ports from physical attacks. Users connecting over these ports should be properly authenticated, and digital records of these connection attempts should be maintained.	E-TD6, E-TD7, E-TD11
E-TD5: Process and Data Analysis	E4, E5, E11, E8, E9	Data Processing	Actuators on the edge may have an effect on the surroundings. The edge may be capable of altering the behavior and security of cars.	E-TD2, E-TD6, E-TD9
E-TD6: Data Storage	E5, E6, E11, E12, E13, E16	Database access	Data storage at the edge will be centralized in a single piece of memory hardware. Due to its exposure to manipulation, it is critical to provide safeguards such as encryption, access control, and authentication to the whole disk to prevent threats.	E-TD3, E-TD4, E-TD5, E-TD9, E-TD10
E-TD7: Energy System	E7	Physical Interaction	Electricity will be used to power edge systems. Alternative energy sources (such as batteries and renewable energy sources such as solar) may be employed in places where supplying electricity is difficult.	E-TD4
E-TD8: Actuators	E8, E10	Physical Interaction	Actuators on the edge may have an effect on the surroundings. The edge may be capable of altering the behavior and security of cars.	Environment (eg. Fire, Radiation, Magnetic waves, Thermal elements), E-TD5, E-TD12
E-TD9: Monitoring	E9, E12	Data Processing	Both the edge and the cloud will need to keep track of their activities. This enables analysts to comprehend why a certain series of events happened. They will also be required to comprehend the system’s performance characteristics.	E-TD5
E-TD10: Sensors	E13, E14	Data Processing and Transmission	The edge is equipped with both internal and exterior sensors. Individual devices inside an edge may have sensors that provide information about the status of the environment within the systems. Meanwhile, external sensors may provide information about the edge of its environment, such as its surroundings.	E-TD6, E-TD12
E-TD11: Devices	E15	Data Processing and Transmission	Smartphones, Bluetooth devices, laptops, and desktop computers are all examples of devices and peripherals. Admins and operators would use these devices to communicate with the edge in order to maintain or operate the system. These are additional methods via which an adversary may breach the system.	E-TD4
E-TD12: Roadside Infrastructures	E10, E14, RV	Physical Interaction, Data Processing, and Transmission	COHDA units are used to represent roadside infrastructure. These devices would be utilized by traffic controllers, CAVs, and other edge devices to carry out ITS activities.	E-TD10, CAVS

**Table A3:** CCAV Reference Architecture—Detailed description of Trust Domains, Data Flow ID, Data Processes, Data Flow Description, Threat Entry/Exit from Figure 2.

CCAV Reference Architecture				
**Trust Domain**	**Data Flow ID**	**Data Process**	**Description**	**Threat Entry/Exit**
C-TD1: Wireless Communication	EC2C, T2C, C1, C2	Data Transmission	Cloud communication presents a significant challenge due to the need for advanced scalability, performance, dependability, durability, and resilience. To achieve optimal results, the cloud must feature a sophisticated architecture consisting of multiple edge clouds interconnected via multiple gateways, operating with maximum efficiency.	Communication Unit (Software and Hardware modules), Antenna, Third-Party Cloud Services, Edge Clouds
C-TD2: Data analysis	C4, C5, C7	Data Processing and Data Transmission	Advanced data analysis in the cloud due to large data volume enables various functionalities such as traffic control and timely distribution. The cloud predicts future trends by evaluating data from edge requests.	C-TD3, C-TD5, C-TD6
C-TD3: Microservices	C1, C3, C4	Data Processing and Data Transmission	The microservices module is responsible for delivering services comprised of multiple individual services. It is renowned for its ability to provide unique services while promoting scalability and ease of testing. For example, intersection management.	C-TD3, C-TD5, C-TD6
C-TD4: APIs	C2, C3	Physical Interaction and Data Transmission	Application Program Interfaces (APIs) are used by users and software modules to obtain access to a specific service.	C-TD1, C-TD3
C-TD5: Data Storage	C6, C7, C8	Data Processing	Edge data storage will be centralized in memory hardware. Given its susceptibility to manipulation-based attacks, it is imperative to implement security measures such as encryption, access control, and authentication to secure the entire disk. The edge’s actuators can impact the environment and have the potential to modify the behavior and security of vehicles.	C-TD2, C-TD3, C-TD7
C-TD6: Monitoring and Logging	C5	Data Processing	Cloud-based decisions made while monitoring the environment, traffic, and other characteristics are saved for future verification. This would allow assessment in the event of a system anomaly or real-world mishap. This is a characteristic of accountability.	C-TD2
C-TD7: Physical I/O	C9	Physical Interaction	Connection to the Cloud infrastructure is made possible via the physical IO ports. Traffic operators connect over these ports to access, update, create, delete, and maintain services. Such personnel should be properly authenticated, and digital records of these connection attempts are to be maintained.	C-TD5, Traffic operator input/output
C-TD8: Energy Systems	C10	Physical Interaction	Cloud data storage is vulnerable to natural disasters, power outages, cyberattacks, and human errors that can cause data loss and breaches. Energy providers must implement security measures, backups, and redundancies with disaster recovery plans while being informed of current threats.	Environment(e.g., fire, radiation, magnetic waves, thermal elements) or insider threats

**Table A4:** CCAV Threats (Red: High-Risk, Yellow: Medium-Risk, Blue: Low-Risk).

Trust Domain	STRIDE	Security Req.	Entry Point	Threat Desc.	Impact	Countermeasures	D	R	E	A	D	Risk
**V-TD1:** Wireless Communication (WiFi, Cellular, 5G/LTE)	S	Authenticity	Wireless Communication (WiFi, 5G/LTE)	Spoofing wireless communication protocol	Changing the code and/or file system of the Wireless Communication Module (compromising integrity) An adversary may interrupt communication, (compromising availability)	The system must be able to detect at run-time that code has been added or changed	3	3	3	4	3	3.2
	D	Availability	Wireless Communication Module (WiFi, 5G/LTE)	Jamming wireless communication channel [65,66,67,68]	An adversary may jam the specific channel or the environment may influence the signals	Jamming detection and prevention techniques such as the following: Channel hopping; Reactive jamming detection techniques, control channel attack prevention; Trigger node identification; Hermes node.	4	4	4	5	4	4.2
	E	Authorization	Wireless Communication Module (WiFi, 5G/LTE)	A malicious adversary/bot may gain additional privileges [69,70,71]	An adversary user may gain privileges to perform network propagation	The system must check if the access rights of the system have any malicious modifications	5	3	1	5	4	3.6
	T, R, I	Confidentiality, Nonrepudiation, Integrity, Privacy	Wireless Communication Module (WiFi, 5G/LTE)	MITM in wireless communication (Frame injection, Data replay, Brute force) [54,60,70,71,72,73,74,75,76]	Running traditional man-in-the-middle attack tools on a suspicious twin node to intercept TCP sessions (compromising confidentiality)	TLS encryption, RADIUS authentication server	5	3	3	4	2	3.4
	E	Authorization	Long-range cellular wireless access [77]	Long-range wireless channels cellular access using voice, 3G: This can be executed by reverse engineering protocol such as AqLink of the onboard telematics system [65] To exploit this flaw, one must first authenticate in order to establish a call timeout value long enough to send a payload of suitable length. Remote access Practical attack [69] Scalability is high	Changes the voice timeout from 12 to 60 seconds, then recalls the automobile and attacks the newly discovered buffer overflow issue. Instructing the car to play a preprogrammed tune using the phone’s microphone	Restrict access	5	2	1	5	4	3.4
**V-TD2**: Infotainment, **V-TD7**: HMI	S, T, I, D, E	Authenticity Integrity Confidentiality and Privacy Integrity Authorization	Primary infotainment ECU (head unit) Telematics Control Unit (T-Box)	Physical access to the head unit through OBD an CAN bus—code accessed through interface or Bus system, internal data to maliciously inject code Later remote access to both head unit and t-box Proximity—physical access with the possibility for remote access Practical attack [66,71,73,78,79,80,81,82,83,84,85,86,87] Scalability is small	Indirect physical channel leading to complete control of vehicle system. Adjust the color of interior LED lights Show photos on the infotainment system [88] Firmware updates [89] Access to CAN bus and other gateways to perform alter electric window lift system, warning lights, airbag control system, and gateway ECUS [31]	Improve code robustness Intrusion detection techniques	5	2	2	3	5	3.4
	T, D	Integrity and Availability	CD Reader	CD-based firmware update—peer-to-peer exchange of media files. Exploitation of firmware present in the media player to execute arbitrary code leading to buffer overflow attack [90] Proximity—physical Access with the possibility for remote access Practical attack Scalability is small	Formatted CD due to which the system can be completely flashed with any adversarial data	Improve code robustness Intrusion detection techniques	3	3	3	3	4	3.2
**V-TD3**: Data Storage	S, T	Authenticity and Integrity	Firmware Firmware Update Debug info. Local Dynamic Map Software [73,79,91]	Inject fabricated frames in memory, e.g., blurry frames, wrong tags Amend firmware to produce fabricated frames leading to the creation and deletion of point cloud or frames Deter/accelerate/delay status and information of modules Inject malicious coordinates to places Replay of frames Byzantine attack Proximity—remote access [37] Simulated attack [37] Scalability is high [37,72,79,82,84,92]	False warnings or services Removed warnings or services Delayed warnings or services	Correlation of messages from neighboring vehicles and cross-verification Restrict access and improve code robustness	5	2	2	3	5	3.4
**V-TD4**: Vehicle Sensors	S, T, E	Authenticity, Integrity, and Authorization	Camera [77]	Bright (250 lx) and dark (0 lx) environments, with different light sources at multiple distances (50 cm, 100 cm, 150 cm, and 200cm), presentation attack [93] Fake env. conditions [36,94] Physical access—close proximity to the vehicular camera Blinding attack [95] Phantom attack Practical attack Scalability is high	Environmental light considering the light wavelength and distance between the cameras leading to incorrect model recognition [95] Not able to tune the autoexposure	Introduction of multiple cameras for redundancy checks A random private signal called a ‘watermark’ could be added to the actuators to detect tampering in sensor measurements	5	5	5	4	5	4.8
	S, T	Authenticity and Integrity	Ultrasonic [95]	Jamming attack may be accomplished by broadcasting ultrasonic noises that overwhelm the membrane on the sensor By adjusting the timing of spoofed pulses, an attacker can manipulate the readings of the sensor Practical attack Scalability is high	Failing to detect obstacles can lead to collisions in parking or maneuvering. Incorrect data sensed can lead to collisions [95]	Introducing multiple sensors for redundancy check Random probing Probing multiple times	5	5	4	4	5	4.6
	S	Authenticity	LIDAR [77,96,97]	Having a working knowledge of LIDAR and a set of transceivers, the attacker receives the LIDAR signal and relays it to the next vehicle. Two transceivers; LUX 3 uses light with a wavelength of 905nm and transceiver B is a photodetector sensitive to this wavelength Practical attack Scalability is high	Incorrect data sensed, which could cause trivial vehicular impacts leading to incorrect model recognition	Introducing cost-effective redundant LIDAR sensors Random probing Probing multiple times Shortening pulse period	5	5	4	4	5	4.6
	D	Availability	Global Navigation Satellite System (GNSS) [98]	Jamming may be accomplished by broadcasting powerful signals that overwhelm the GPS receiver Practical attack Remote access Scalability is high	Incapable of detecting original signals	A large number of GPS receiver modules lack robust antijamming protection. Numerous companies have built equipment to identify interfering signals. However, these defense tactics may not be effective against CAVs due to their dynamic movements [98] Adaptive array technologies [99] Turbo coding methods for counter jamming [100]	4	3	4	4	4	3.8
	S	Authenticity and Integrity	Global Navigation Satellite System (GNSS) [98]	An attacker transmits erroneous yet acceptable GPS signals in order to fool GPS receivers on CAVs. Attacks begin by sending signals similar to those sent by legitimate satellites. They then progressively boost the strength of their transmissions and deflect their GPS signals from the genuine position of the target [101,102]. Practical attack [103,104,105] Remote Access Scalability is high	GPS device processes counterfeited signal	Monitor GPS signal strength: average or compared between time frames [106] Monitor the signal strength of each satellite transmission received [106] Satellite identifying codes and the quantity of received satellite signals are monitored [106] Time interval comparison and verification [106] Sanity check [106]	4	3	4	4	3	3.6
	S, T, I, E	Authenticity, Integrity, Confidentiality, Privacy, and Authorization	Auxiliary Sensors: Vehicle’s custom telematics features such as UConnect. This includes onboard connectivity features using wireless sensors and CAN bus vulnerabilities	Remote access to vehicle communication systems with the ability to flash the firmware version [26] Availability of Uconnect’s DBus Port to be open for communication Remote access Practical attack [87,92,107] Scalability is high	Incorrect data sensed, which could cause trivial vehicular impacts leading to incorrect model recognition	Code robustness Restricted Access to OBD and patching vulnerable software	3	3	1	3	3	2.6
**V-TD5**: Physical Input/Outputs	T, I	Integrity, Confidentiality, and Privacy	Onboard Diagnostic Port [108,109] USB	Replay collected CAN packets, capturing each CAV response. The modified CAN packets might then control the vehicle’s behavior. This is enabled with access to the OBD port and with a Windows PC operating system capable of analyzing CAN packets [110] Direct physical access BUS attack Practical attack Scalability is small Firmware tampering [91]	Horn: Raise the horn continuously Vehicle brakes: Slam brakes at any speed Gas: Change speedometer and gas gauze at will Engine: Cause engine to accelerate Battery: Prevent the car from powering down and/or draining the battery Disable seat belt notification Disable power steering or jerk wheel Turn headlights on or off when left in automode	Code robustness Restricted access to OBD port	5	4	4	3	4	4
**V-TD6**: Monitoring	E	Authorization	White-box and black-box attack	Adversarial input models are more effective at producing successful mispredictions of signboards at a quicker pace and with a larger likelihood of failure[111] Remote access Practical lab-based attack Scalability is high	Mispredictions of signboards	Intrusion detection system with better machine learning algorithms that could predict images based on [94]: - Size - Angle - Focus - Context - Surface - Lightning - Depth	4	3	2	2	3	2.8
**V-TD8**: Energy System	D	Availability	Energy and fuel storage, power generation	Directed energy weapons Electronic warfare Adjust charging current [112]	Directed energy is able to operate as a force multiplier without visual signs or detection. As a result, it can ultimately damage the targeted unit It includes jamming and spoofing to control the electromagnetic spectrum. Uplink jamming can be directed toward the satellite and space-orbiting vehicles, which can impair the services for all users in the satellite reception area. Spoofing deceives the receiver by introducing a fake signal with erroneous information Other systems can deliver temporary or permanent effects against the vehicles by using a radio frequency jammer, lasers, chemical sprayers, and high-power microwaves. It can cause damage to the vehicle	It is useful to shield a CAV system with a material with reflective and thermal properties. If the engagement can be detected, the satellite or space orbiting vehicle can use electronic systems to jam the terminal guidance of the electronic warfare weapon Deployment of attack sensors Development or maintenance of electronic countermeasures and electro-optical countermeasures	5	4	1	5	5	4
**V-TD9**: Actuators	S, T, D, E	Authenticity, Integrity, Availability, and Authorization	Body Control Module (BCM)	Device control packet manipulation using fuzzing and sniffer. Packet sniffing and targeted probing on a car Access to CAN bus network Practical attack [65,70,79,113] Scalability is small	The control of all vehicular body parts in motion, such as the following: - Continuous activation of lock relay; - Activation of windshield wipers; - Trunk unlocking; - Unlocking doors; - Permanent activation of the horn; - Disabling and enabling of headlights and auxiliary lights; - Release of wiper fluid; - Control of horn frequency; - Control of instrument brightness; - Physical access; - Practical attack; - Scalability is high.	Code robustness Restricted access to OBD port	5	1	1	3	5	3
	S, T, D, E	Authenticity, Integrity, Availability, and Authorization	Electronic Control Module (ECM)	Device control packet manipulation using fuzzing and sniffer Packet sniffing and targeted probing on a car Access to CAN bus network [114,115] Practical attack [65,70,79,83,113] Scalability is small	Initiate crankshaft or disturb engine timing by resetting the learned crankshaft angle through sensor errors Temporary increase/ boost idle RPM Disable cylinders temporarily, power steering/brakes Kill engine Disable the engine such that it knocks excessively when restarted, or cannot be restarted at all Grind start	Code robustness Restricted access to OBD port	5	1	1	3	5	3
	S, T, D, E	Authenticity, Integrity, Availability, and Authorization	Electronic Brake Control Module (EBCM)	Device control packet manipulation using fuzzing and sniffer Packet sniffing and targeted probing on a car Access to the CAN bus network Practical attack [65,70,79] Scalability is small	Lock of individual brakes without unlocking EBCM Engages front left brake Engages front right brake/unlocks front left brake Unevenly engages right brakes Releases brakes, prevents braking	Code robustness Restricted access to OBD port	5	1	1	3	5	3
	S, T, D, E	Authenticity, Integrity, Availability, and Authorization	Autolock feature for doors, trunk, charging port, and fuel lid—passive keyless entry system—key fob [116]	Replay over the Cable attack: Relay of low-frequency and ultra-high-frequency signals through the generation of magnetic fields to trigger the key fob which demodulates and recovers the original message from the vehicle. Two antennas and an amplifier. Antennas near the door handle capture the beacon signal as a magnetic field locally. This is sent to the other end of the antenna through the amplifier, where it is amplified to increase the signal quality. When this signal reaches the other end, it creates a magnetic field in a second antenna. The PKES would demodulate and message the automobile. Remote access Practical attack [65,68,70,79,117,118,119,120,121,122,123,124,125,126,127,128,129,130] Scalability is high	Passive keyless entry systems compromised Vehicle unlocking Ignition system	Immediate countermeasures: shielding the key, and removing the battery from the key Midterm countermeasures: software-only modification, access control restriction, and hardware modification	5	3	3	4	5	4
	E	Authorization	Autolock doors and trunk—passive keyless entry system—key fob [77]	Replay over-the-air attack: RF links with emitter and receiver to receive, amplify, and transmit the signals from the car to the PKES. The emitter amplifies and transmits the vehicle’s RF signals at 2.5 GHz. The vehicle’s receiver gets the signal and converts it down to LF. Once the key fob reacts, the vehicle doors and even the engine can be unlocked. Remote access Practical attack Scalability is high	Passive keyless entry systems compromised Vehicle unlocking Ignition system	Immediate countermeasures: shielding the key, removing the battery from the key Midterm countermeasures: software-only modification, access control restriction, and hardware modification	5	3	3	4	5	4
**V-TD10**: Data Analysis	R, I	Nonrepudiation, Confidentiality, and Privacy	Cooperative Awareness Message (CAM) [37]	Malicious advertisers(V2V/V2I) generate congestion response messages based on the content of the congestion requests [54,60,75,76] Proximity—remote access [37] Simulated attack [37] Scalability is high [37]	The overall speed of CAVs could be affected. Increase in traffic in the targeted or neighboring roads. Parked bots or compromised vehicles could be infected	Correlation of messages from neighboring vehicles and cross-verification	5	4	4	5	3	4.2
	T, R, I, E	Integrity, Nonrepudiation, Confidentiality, Privacy, and Authorization	Cooperative cruise control module Localization Vehicle control warning, e.g., lane departure warning Vehicle intersection warning Object identification Vehicle control automation Sensor fusion	Fake env. conditions for camera lens [94] Inject fabricated frames directly into the camera processing and memory Infect camera firmware in order to generate fabricated frames indicating unintentional activities such as lane departure Frames are modified with property changes such as blurry images to confuse modeling software Insert fabricated frameworks and graphic models to indicate warnings Remove frames that indicate normal or abnormal conditions Deter/rush/delay delivery of speed, steering wheel info., status Inject code that processes frames and generates models or alters internal memory with fabricated frames [54,60,75,76] Inject code to fuse sensed data to indicate warnings and malicious outputs [71,94,105,131]	Compromised cooperative cruise control functionalities leading to the following: - An accident; - The inability to activate functions; - Driving into traffic; - Discomfort. The following are the subimpacts: - Sensor information altered; - Sensor preprocessor manipulated; - Main processor manipulated; - Audio system exposed; - HMI is vulnerable; - Low-level controllers influenced; - Vehicle status altered; - Road and environmental condition prediction modules influenced; - Altered video frames, graphic models; - Altered vehicle dynamics information.	Conserve functional simplicity Securing real-time analytical software and operating system Zero-trust architecture Multi-interface security and isolation [24]	5	4	3	5	4	4.2
**V-TD11**: Devices and Peripherals	D, E	Availability and Authorization	Bluetooth-enabled smartphone devices [77,132] Company-owned proprietary devices	Indirect Bluetooth access: vulnerability present in the custom interface code of the Bluetooth-enabled telematics system. This requires pairing an adversarial device to the vehicle’s Bluetooth Direct Bluetooth access: vulnerability present in the custom interface code of the Bluetooth-enabled telematics system. This requires pairing an adversarial device to the vehicle’s Bluetooth Execution of any arbitrary code and taking control of the entire Vehicular systems Remote access Physical access [81] Practical attack [69,80,122,133,134] Scalability is small	Execution of any arbitrary code and taking control of the entire vehicular systems by access to program handling Bluetooth functionality Compromise of the telematics ECU’s Unix operating system Exfiltration of data	Restrict access	5	3	3	3	3	3.4
	S, T, I	Authenticity, Integrity, Confidentiality, and Privacy	User devices (insider, guest, bring-your-own-device for employees) [77] Mobile applications	Information injection: device controlled by an adversary can inject malicious code or information [135] Service manipulation: virtual machines could be manipulated Information disclosure of vehicles and RSUs [92,112,120,136,137]	Execution of any arbitrary code and taking control of the entire Vehicular systems by access to program handling Bluetooth functionality Compromise of the telematics ECU’s Unix operating system Exfiltration of data	Restrict access Anomaly detection techniques	3	4	3	3	3	3.2

**Table A5:** Edge/Cloud and Cloud Threats (Red: High-Risk, Yellow: Medium-Risk, Blue: Low-Risk).

Trust Domain	STRIDE	Security Req.	Entry Point	Threat Desc.	Impact	Countermeasures	D	R	E	A	D	Risk
**E-TD1, C-TD1** Edge/Cloud Communication (Wifi, Cellular, 5G/LTE)	D	Availability	Wireless communication (WiFi, 5G/LTE)	Denial of Service and distributed DoS by wireless jamming	Disrupt the vicinity of the impacted network Channel hopping Reactive jamming detection techniques, control channel attack prevention Trigger node identification	Protocols and services can be designed to operate in an autonomous or semiautonomous manner	3	3	3	4	3	3.2
	S, T, I	Authenticity Integrity Confidentiality Privacy	Wireless Communication Module (WiFi, 5G/LTE)	Adversaries can launch attacks such as eavesdropping and/or traffic injection Public network IP [66,78]	Gateway compromised and access to internal network interfaces. Dangerous attack	Jamming detection and prevention techniques, such as the following: Network segmentation; The system must check if the access rights of the system have any malicious modifications; TLS encryption; RADIUS authentication server.	4	3	1	3	3	2.8
	E	Authorization	Wireless Communication Module (Wifi, 5G/LTE)	Rogue gateway: open system where any devices can be part of the system	An adversary may gain privileges to propagate the network and create personal cloudlets	The system must check if the access rights of the system have any malicious modifications	5	2	1	4	2	2.4
**E-TD2, C-TD3**: Microservices	D	Availability	Wireless communication, virtualization servers	Physical damage: the systems may not be guarded as they may be managed by the service provider	The impacts of both attacks are limited to the local vicinity and scope The impact can be even worse as they can extract sensitive information about the users in the location due to contextual awareness	Identity management and authentication Protocol and network security Code robustness Restricted access Distributing services and migrating virtual machines	5	3	2	5	1	3.2
	I	Confidentiality Privacy		Privacy leakage: internal threats and honest but curious actors may attempt to access the information [66,78]			4	4	2	4	5	3.8
	E	Authorization		Privilege escalation: infrastructure could be misconfigured			4	3	2	4	2	3
	T, I	Integrity Confidentiality Privacy		Service manipulation such as amending the functions of a CAV application such as forming, joining, leaving, merging, splitting, ending, and changing leader Covert channel attack Black/gray hole attack			5	2	2	5	2	3.2
**E-TD3, C-TD4**: API	I	Confidentiality Privacy	Local infrastructure interface, vehicle-to-car interfaces	Privacy leakage: internal threats and honest but curious actors may attempt to access the information [66,68,78,104]	The impacts of both attacks are limited to the local vicinity and scope In some cases, the distributed services and migrating virtual machines The impact can be even worse as they can extract sensitive information about the users in the location due to contextual awareness	Identity management and authentication Protocol and network security Code robustness Restricted access	3	4	3	4	5	3.8
	E	Authorization		Privilege escalation: infrastructure could be misconfigured			4	3	2	4	2	3
	T, I	Integrity Confidentiality Privacy		Service manipulation such as amending the functions of a CAV application [138]			5	2	2	5	2	3.2
	T, I	Integrity Confidentiality Privacy		Rogue services			5	2	2	5	2	3.2
**E-TD4, C-TD7**: Physical Input/Outputs	T, R, I, E	Integrity Confidentiality Privacy Nonrepudiation Availability Authorization	Edge ports with devices and peripherals	Direct physical access through connected devices Practical attack Scalability is small	Disrupt the edge Inject false messages Interrupt the functioning of the edge Exfiltrate data	Identity management and authentication Protocol and network security Network segmentation Zero-trust architecture Code robustness Restricted access	5	5	4	5	3	4.4
**E-TD5, C-TD2**: Edge Process and Data Analysis	T, R	Integrity, Nonrepudiation	Wrong data, protocols and data from communication, microservices, and data storage module [54,75]	Inject fake env. conditions for edges [94] Inject fabricated data directly in edge processing and memory Infect firmware in order to generate fabricated data indicating unintentional activities such as lane departure Frames are modified Remove data that indicate normal or abnormal conditions Deter/rush/delay delivery of data Inject code that processes frames and generates models or alters internal memory with fabricated frames Inject code to fuse sensed data to indicate warnings and malicious outputs [81,139,140]	Midlevel vehicle function optimizer, Local Dynamic Map, security management, edge platform management The overall information for CAVs could be affected. This can lead to a joint increase in traffic on the targeted or neighboring roads. Parked bots or compromised vehicles could be infected Leads to traffic Leads to discomfort	Correlation of messages from neighboring vehicles and cross-verification Conserve functional simplicity Securing real-time analytical software and operating system Zero-trust architecture Multi-interface security and isolation [24] Code robustness and testing	4	3	3	5	3	3.6
**E-TD6, C-TD5**: Data Storage	T, I	Integrity Confidentiality Privacy	Firmware Firmware update Debug info. Local Dynamic Map System-level information Software	Inject fabricated frames in memory, e.g., blurry frames, wrong tags Amend firmware to produce fabricated frames leading to the creation and deletion of point cloud or frames Deter/accelerate/delay status and information of modules Inject malicious coordinates to places Remote access with/without cables for information loss [136,141,142] Replay of frames Delete/reveal the system software or data [78] Proximity—remote access [37] Simulated attack [37,143,144,145,146,147,148,149,150] Scalability is high [37]	False warnings or services Removed warnings or services Delayed warnings or services	Correlation of messages from neighboring vehicles and cross-verification Restrict access and improve code robustness	5	3	3	5	3	3.8
**E-TD7, CTD8**: Energy System	D	Availability	Energy and Fuel Storage, Power Generation	Energy and fuel storage, power generation and directed energy weapons Electronic warfare Kinetic energy threats	Directed energy is able to operate as a force multiplier without visual signs or detection. As a result, it can ultimately damage the targeted unit and cause losses. It includes jamming and spoofing to control the electromagnetic spectrum. Uplink jamming can be directed toward the satellite and space-orbiting vehicles, which can impair the services for all users in the satellite reception area. Spoofing deceives the receiver by introducing a fake signal with erroneous information.	Protect and shield energy system using reflectance techniques with thermal qualities, practices with disaster recovery techniques If the engagement can be detected, the systems can be protected using electronic warfare weapon Deployment of attack sensors Deployment of a surveillance system Development or maintenance of electronic countermeasures and electro-optical countermeasures	5	5	5	5	5	5
**E-TD8**: Actuators	D	Availability	Edge processing and physical access	Manipulate actuators Disable actuators	The control of all edge-based actors Manipulate or disable barriers Manipulate or disable traffic signal	Code robustness Restricted access	4	3	2	4	4	3.4
**E-TD9, C-TD6**: Monitoring	E	Authorization	White-box and black-box attack	The adversarial model is more effective at producing successful mispredictions of signboards at a quicker pace and with a larger likelihood of failure [111] - Remote access - Theoretical attack - Scalability is high	Mispredictions of signboards	Intrusion detection system with better machine learning algorithms that could predict images based on the following [94]: - Size; - Angle; - Focus; - Context; - Surface; - Lightning; - Depth.	3	2	3	4	2	2.8
**E-TD10**: Edge Sensors	S, T, D	Authenticity Integrity Availability	Internal sensors which relies on the network layer [151]	Jamming attack Timing attack Replay attack Routing threats	Uniform coding Conflict collision Privacy disclosure Redundant sensors with integrity checks Sensor fusion Attack detection techniques Noise filters Machine-learning-based solutions	Zero-trust architecture Deperimeterization Software-defined perimeter	3	3	3	3	2	2.8
	S, T, R, D, E	Authenticity Integrity Nonrepudiation Availability Authorization	External sensors include rain sensors, pH sensors, smart meters, temperature sensors, humidity sensors, sound sensors, vibration sensors, chemical sensors, and pressure sensors [151]	Tampering Sensor device capture Fake device and malicious data Sybil attack Source device authentication problem Implicit deduction from sensor behavior Encryption leakage			4	4	3	4	3	3.4
**E-TD11**: Devices	T, I	Integrity Confidentiality Privacy	User devices (insider, guest, bring-your-own-device for employees) [77]	Information injection: device controlled by an adversary can inject malicious code or information [135] Service manipulation: virtual machines could be manipulated Information disclosure of vehicles and RSUs [137,141]	Execution of any arbitrary code and taking control of the entire vehicular systems by access to program handling Bluetooth functionality Compromise of the telematics ECU’s Unix operating system Exfiltration of data	Restrict access Anomaly detection techniques	3	4	3	3	3	3.2
**E-TD12**: Roadside Infrastructure	E	Authorization	These devices could be end-notes such as COHDA units or internet-of-things devices	Wired connections could be manipulated to connect with rogue systems to give feedback to edge systems with artificially coded messages Execution of any arbitrary code and taking control of the RSUs Remote access Practical attack [81,133,134,139,140,152,153] Scalability is high	Execution of any arbitrary code and taking control the systems by access to programs Compromise the operating system Exfiltration of data Analysis of the current contextual awareness	Restrict access Anomaly detection techniques	4	4	3	3	4	3.6
